# Making a Place for Space: A Demographic Spatial Perspective on Living Arrangements Among the Elderly in Historical Europe

**DOI:** 10.1007/s10680-019-09520-5

**Published:** 2019-03-21

**Authors:** Mikołaj Szołtysek, Bartosz Ogórek, Radosław Poniat, Siegfried Gruber

**Affiliations:** 1 Institute of History, University of Warsaw, Krakowskie Przedmieście 26/28, 00-927 Warsaw, Poland; 2 Institute of History and Archival Science, Pedagogical University of Cracow, Podchorążych 2, 30-084 Cracow, Poland; 3 University of Białystok, Plac Niezależnego Zrzeszenia Studentów 1, 15-420 Białystok, Poland; 4 Karl-Franzens-Universität Graz, Mozartgasse 3, 8010 Graz, Austria

**Keywords:** Ageing, Living arrangements, Spatial autocorrelation, Family systems, Census microdata, Intergenerational co-residence, Local Indicators of Spatial Association

## Abstract

Much of the previous scholarship on the historical living arrangements of the aged has taken place without the benefit of large-scale harmonised census microdata and did not embrace even rudimentary forms of spatial modelling. Drawing on the pooled cross-sectional census microdata from the North Atlantic Population and Mosaic projects, we derive measures of intergenerational co-residence among the elderly for 277 regional populations from Catalonia to the Urals during the demographic ancien régime and thereafter. To examine the historical geography of living arrangements among the elderly, the spatial patterns in our data are assessed using formal tools of Exploratory Spatial Data Analysis. To investigate the extent to which the observed regional patterns are attributable to underlying demographic, socio-economic, or environmental variability, we specified a series of the OLS regression models and applied the Local Indicators of Spatial Association to the models’ residuals in order to identify the spatial clusters that cannot be explained by the chosen set of predictors. Our findings reveal considerable variability in the living arrangements of the elderly in historic Europe. This variability does not align very neatly with the geographic patterns predicted by earlier historical demographic literature and partly persists even after controlling for contextual factors. Our bottom-line results suggest that when seeking to untangle the dynamics of European family systems, greater spatial awareness is indispensable.

**Electronic supplementary material:**

The online version of this article (10.1007/s10680-019-09520-5) contains supplementary material, which is available to authorized users.

## Introduction

During the last 3 decades, there has been a surge of interest in the living arrangements of older people in the past. The burgeoning literature on this topic has revealed that the co-residence patterns of the aged varied considerably between European societies (e.g. Smith 1981; Laslett 1988; Pelling and Smith 1991; Andorka 1995; Kertzer and Laslett 1995; Ruggles 1996a; Wall 1984, 1995; Alter 1996; Alter et al. 1996; Fauve-Chamoux 1996; Reher 1998; recently, Szołtysek and Gruber 2014; Magnuson 2016; Kuklo 2018)^1^. However, few attempts have been made to measure and understand these patterns systematically across space. Robust comparisons have been hampered by limited data availability, selective methodologies, and significant gaps in the evidence. Earlier attempts to study this topic were also hindered by the failure to account for the effects of demography on co-residence patterns (cf. Ruggles 2012). Last but not least, despite continuing interest in the geography of European family forms (e.g. Ruggles 2010; Alter 2013; Moring 2016; Micheli 2018) and the rapid advances in the technology of spatial data handling (Gutmann et al. 2011), there has, to date, been no historical research on elderly living arrangements that used emerging geospatial data or even rudimentary forms of spatial modelling.

This paper expands on the existing literature in three major ways. First, it combines data from the North Atlantic Population Project and the Mosaic project to create a Europe-wide historical database of an unprecedented scope. Second, it uses formal inductive methods of spatial analysis rather than the crude geographic typologies that have heretofore dominated historical studies of family structure. Third, it accounts for the effects of contextual variation across populations by introducing local-level control variables within a multivariate regression framework. These three methodological pillars allow us to probe a number of questions that have rarely been asked before: What does the geography of elderly living arrangements look like when we examine large-scale historical evidence using the formal tools of spatial analysis? Was this geography generated by structural stability over space, or was it conditional on local spatial heterogeneity? Does the spatial patterning revealed in a uniquely broad set of comparative materials reaffirm the received wisdom that the family contexts of the elderly differed between major areas of Europe, and how sensitive is that picture to the effects of potential determinants of co-residence patterns?

Our findings have uncovered significant spatial dynamics in the living arrangements of the elderly in historical Europe that are embodied in two main spatial properties of our data. First, there are strong tendencies for regions or sets of regions with similar patterns to cluster close to each other, which indicates that the underlying co-residence order among the elderly was spatially dependent. However, within this global structure, discrete local or regional spatial regimes based on similarity can be identified whose presence does not align neatly with the geographic patterns predicted by earlier literature. Although a large share of the global and local variation across our data is driven by demographic, socio-economic, and environmental conditions, some of the regional specificity of the living arrangements of the elderly persists even after controlling for these factors. Our bottom-line results suggest that when seeking to untangle the dynamics of European family systems, greater spatial awareness is indispensable.

## Background

Following Le Play, many scholars have argued that for most periods of human history, European family systems were not randomly distributed geographically. Starting with the seminal volumes by Laslett and Wall (1972) and Wall and Robin (1983), historians of the European family have juxtaposed the complex household formation system of major Eurasian societies with the north-western European system based on small nuclear families (e.g. Hajnal 1983; Engelen and Wolf 2005; Fauve-Chamoux and Ochiai 2009). Around this mainstream classification based on the morphology of household systems a rigid geographic taxonomy of family systems has been built up that divides Europe into three parts separated by two main boundary lines, splitting north-western Europe from the south and the western from the eastern part of the continent.

While the exact spatiotemporal location of these frontiers continued to be debated, there was general agreement that the basic parameters of family life among the aged were shaped very differently by differently configured family systems. The standard view among scholars was that the two disparate models of household formation in historical Europe (Hajnal 1983), the nuclear family model and the complex (joint and stem) family model, reflected contrasting systems of intergenerational support, and that in the latter model, the care of the elderly fell almost exclusively on the family, whether by means of co-residence or the circulation of the elderly among the households of their offspring and other relatives (e.g. Laslett 1988; Hammel 1995; also Reher 1998). By the end of the 1990s, it was clear that this distinction was too simplistic, and that the geographic distribution of the historical living arrangements of the aged was too patchy to allow any rigid divisions of the continent to be drawn (e.g. Pelling and Smith 1991; Kertzer 1989; 1991; Wall 1991; also Manfredini and Breschi 2013). However, an effective investigation of this conundrum required the use of data that did not yet exist and of methodologies that were beyond the reach of family historians at that time. The vast majority of quantitative research on the historical co-residence patterns of the aged that had been conducted up to that point consisted of studies of a single community or a small group of communities and relied on a range of unharmonised approaches. Thus, the data from these studies were not systematically comparable and posed further challenges of the microlevel stochastic variations (e.g. Johansen 1976; Smith 1981; Laslett 1983, 1988; Rose 1988; Zitomersky 1987; Kertzer and Laslett 1995; Gunnlaugsson and Garðarsdóttir 1995; Wall 1984; Laslett 1989; Alter 1996; cf. Szołtysek and Gruber 2014). For many areas of Europe, hardly any data were collected, and these gaps in the evidence made advancing spatial models very difficult. Finally, although family historians were good at documenting local specificities of elderly co-residence patterns, they failed in their efforts to systematically untangle the causes of these patterns and remained oblivious to their potential demographic underpinnings (cf. Wachter et al. 1978; Ruggles 1987).

With the advent of the information revolution in historical population studies in the 2000s (Ruggles 2012; Ruggles et al. 2011), these practices changed dramatically, empowering researchers to analyse multiple censuses as a single data set and thus enabling them to measure the living arrangements of the elderly across multiple settings. In two pathbreaking studies, Ruggles (2009, 2010) compared the co-residential patterns of the aged based on data from 87 censuses (including historical censuses) carried out in 34 countries. While Ruggles’ comparative analyses remain laudable achievements, as his data did not include evidence from pre-1980 continental Europe or from Eastern Europe, he was unable to provide a truly European overview of the living arrangements of the elderly (cf. Gruber and Szołtysek 2012).

Another recurrent problem that can be observed in all subsequent works on historical co-residence patterns among the elderly is the failure to use an explicit spatially sensitive approach. Although space has been a central organising concept in many historical demographic works on family systems, the unsystematic character of most of the historical evidence hindered efforts to analyse the spatial dynamics of the living arrangements of the elderly. Instead, students of historical family variation had to rely on simplified and often ahistorical classifications of family systems based on global geographic regions and to use crude implicit geographic macro-level models as major heuristic devices (Hajnal 1983; Dennison and Ogilvie 2014; Reher 1998; Therborn 2004; Todd 2011). Although Ruggles (2009, 2010) acknowledged that the co-residence patterns of the elderly could differ across the geographic regions of his worldwide sample, the empirical design of his studies was nevertheless *aspatial* (cf. Fotheringham and Rogerson 1993, 4), as aggregate national census samples largely precluded spatial analysis in any formal sense.

The recent emergence of the large, georeferenced, historical databases we use in this paper (see below) has made it possible to investigate more explicitly the local spatial patterns of the living arrangements of the elderly and thus to take advantage of the analytical methods of quantitative geographers, which are increasingly being used to identify and understand spatial variability (Anselin 1995; Fotheringham 1997; Boots 2002; also Gutmann et al. 2011, 2). Paying attention to local spatial effects and relationships—e.g. local patterns of association and local instabilities in the overall spatial associations—may help us to better address the issue of the regionalisation of the living arrangements of the elderly by enabling us to identify areas with similar values for one or several indicators; to locate boundaries between areas and areas with anomalous values within regions; and to identify local patterns that deviate from regional patterns (cf. Unwin and Unwin 1998; Fotheringham 1997). Finally, looking at local spatial patterns may enable us to formally assess the null hypothesis of spatial randomness in the patterns of co-residence among the elderly across Europe; i.e. to determine whether the spatial variation in these patterns was decidedly different than it would have been if governed by chance (cf. Anselin 1995). Accordingly, the comparison of these findings with speculations based on the more fragmentary data that have dominated the literature can be advanced.

## Empirical Methodology

### Data Description

This paper uses data from the combined North Atlantic Population Project (NAPP) and Mosaic databases of historical census microdata, which represent the largest data infrastructure of this kind that has ever existed in Europe (Ruggles et al. 2011; Szołtysek and Gruber 2016; Szoltysek et al. 2017; see Electronic Supplementary Material 1 for the list of all Mosaic/NAPP data sets).^2^ These data are broadly available in the form of machine-readable, harmonised microdata samples derived from various kinds of historical census and census-like materials, including full-count national censuses (NAPP), as well as local/regional fragments of censuses, church lists of parishioners, tax lists, and local estate inventories (Mosaic).^3^

In this study, we use the Mosaic samples of 903,180 individuals living in 126 geographic areas ranging from Catalonia in the west to the Urals in the east that were collected between 1700 and 1918. The NAPP samples expand the collection to Great Britain and Scandinavia, bringing in data for 151 additional historical regions from five national censuses that cover more than 14 million individuals. All of the Mosaic and NAPP samples have a similar structure. Each sample describes the characteristics of all of the individuals in a given locality grouped into co-resident domestic groups and provides a core set of common variables, including information on each person’s relationship to the household head, age, sex, and marital status. These data are harmonised across space and time using the international coding structure of IPUMS, thereby facilitating the creation of a set of dyadic-pointer variables that identify the location within the household of each older individual’s own children, children-in-law, and other relatives (Szołtysek and Gruber 2016; cf. Sobek and Kennedy 2009).

Our approach is situated at the mesolevel of comparative analysis, and our units of analysis are “regions”. The regions in the NAPP data are the administrative units that were used in the respective census and that were considered by the NAPP. The Mosaic data are organised by separate locations, which in most cases also represent separate administrative units.^4^ The combined database includes information on 277 regional populations with 15.1 million individuals living in more than three million households. Of the 277 regional data sets, 59% refer to populations after 1850, while 41% cover populations before 1850, and 21% cover populations that predate 1800. The collection includes information on both rural and urban sites (although rural societies predominate)^5^ and covers large shares of the environmental, socio-economic, cultural, and demographic variation found across Europe. Throughout the paper, the regional data are presented as pooled time cross sections based on the tacit assumption that the family behaviours they pertain to represent “deep” cultural layers that move slowly over time (see Todd 1985; Reher 1998; Therborn 2004; Daatland et al. 2011; Wall 2002; Szołtysek and Poniat 2018b; Schürer et al. 2019). All of these data are georeferenced, which allows us to link them with various GIS-derived covariates and other locational attributes.

Whereas the NAPP data consist of either full-count census data or representative samples taken from them, the Mosaic samples have varying levels of representativeness. The Mosaic samples cover 22 European countries, and most of these data—except for the Croatian, Bulgarian, Belgian, Turkish, and Spanish data—are derived from census microdata covering very large populations from multiple locations and broad geographic areas and thus provide fairly reasonable representations of historical familial diversity in those areas (Szołtysek and Poniat 2018a; also Szołtysek and Gruber 2016, 42–47). Although the combined Mosaic/NAPP data are larger in scope and in coverage than all of the preceding efforts to create a family history data infrastructure, some areas are not yet included. For example, we have no data on Italy and the Iberian peninsula (except Catalonia), where we would likely find a wide range of living arrangements among the elderly (Barbagli 1991).^6^ This gap in the data constrains our ability to explore the north–south dimension of variation in family systems across Europe, as has been discussed, for example, by Reher (1998).

### Measures of Living Arrangements

Our measures of the living arrangements of the elderly are based on definitions suggested by Ruggles, with some modifications (Ruggles 2009, 2010; cf. Gruber and Szołtysek 2012). Following Ruggles, the elderly population is defined as persons aged 65 or older.^7^ Unlike Ruggles, however, married couples in which both partners were aged 65 or older are not treated as single observations, even though they shared a single living arrangement.

We use three indicators of elderly co-residence, each computed as the regional proportion of elderly persons living with certain configurations of kin (or lack of thereof) in the household (see Table 1). First, following Ruggles (2010) and Gruber and Szołtysek (2012), two measures of familial complexity in the historic living arrangements of the elderly were constructed. The variable “living with one ever-married descendant” (henceforth, LMD) captures patterns of intergenerational co-residence that are most similar to stem-family arrangements. Our second variable, “living with 2+ ever-married descendants or ever-married lateral kin” (henceforth, LLK), measures the proportion of elderly persons living in residential configurations that most closely resemble the laterally extended multiple-family domestic groups (joint families) described by family historians and social anthropologists (Szołtysek and Gruber 2014). Finally, the variable “living without any relatives” (henceforth, LWR) captures the residential isolation of the elderly and thus accounts for the prevalence of the nuclear hardship areas in Europe (Laslett 1988).^8^ The construction of all three variables is very much akin to the so-called egocentric approach to mapping family constellations (Hagestad 2000), whereby each elderly person in the census serves as an anchor, and the descending and horizontal relationships are analysed from his or her position (cf. Sobek and Kennedy 2009).

**Table 1:** Definitions of intergenerational co-residence measures

Name (abbrev.)	Definition	Reference	Specification
Elderly persons living with one ever-married descendant (LMD)	Proportion of elderly persons (65 +) living with one ever-married descendant in the child and/or the grandchild generation	Stem-family systems	Descendants include child, child-in-law, grandchild, and grandchild-in-law Nonbiological children and grandchildren (step, adopted, or fostered) are treated as biological descendants Ever-married includes the married, widowed, separated, and divorced marital status Only family households
Elderly persons with 2 + ever-married descendants or ever-married lateral kin (LLK)	Proportion of elderly persons (65 +) living with at least one ever-married lateral relative or widowed lateral relative-in-law, or at least two ever-married descendants (or in-laws) in the child or the grandchild generation	Joint-family systems	Descendants include child, child-in-law, grandchild, and grandchild-in-law Lateral relatives include in-laws Nonbiological children and grandchildren (step, adopted, or fostered) are treated as biological descendants Ever-married includes the married, widowed, separated, and divorced marital status Widowed includes the widowed, separated, and divorced marital status Only family households
Elderly persons living without any relatives (LWR)	Proportion of elderly people (65 +) living without any relatives, not even a spouse	Nuclear (neolocal)-family systems	Applies to all households

The number of living children of older adults is not known because information on children (and other kin) living outside the household is not provided. If there are nonco-resident children, their proximity to the older adult is also unknown

Some inevitable limitations of our measures need to be mentioned. First, given that relatives living outside the household are not mentioned in our data, our measures are not a sufficient indicator of the absolute strength or lack of kinship and intergenerational ties. However, they do provide a strong gauge of the *potential* intra-household support for the elderly (Boele et al. 2018, 362; cf. Grundy 1992, 353; Glaser 1997; Gaymu et al. 2006, 242; Michielin and Mulder 2007, 655). During our study period, such support was crucial to the well-being of the elderly throughout Europe, as domestic groups were the main institutions responsible for the distribution of goods and services between generations (e.g. Szołtysek 2015; also Bongaarts and Zimmer 2002, 145–146). Second, it cannot be completely ruled out that some elderly individuals who were recorded as lodgers or inmates in the listings were in fact “hidden” relatives of the head or another person in the household. However, to the best of our knowledge, such cases would have been sporadic and random (Szołtysek 2015, 818–820; Schürer et al. 2019). The potential under-registration of children less than 1 year old would have biased our estimates only minimally, as very few elderly people would have been co-residing with such young offspring, and most of these children would have been never married.

### Methods

The analysis is carried out in four interrelated steps. We first map the distributions of the focal measures of living arrangements using choropleth maps and box and whisker plots. Given that a visual inspection of distribution maps may be unreliable (Gutmann et al. 2011, 8), in the next step spatial patterns in our data are assessed using formal Exploratory Spatial Data Analysis (ESDA) tools (Anselin 1995). One of the challenges that can arise when using ESDA tools lies in properly defining a network structure that reflects the idea of locality and connectivity (Anselin 1988; Fotheringham and Wong 1991; Griffith 1996). Because of the spatial dispersion of our data points and their unequal density across broader areas of Europe, the five-nearest neighbours network structure (based on the great-circle distances) with a row-standardised inverse distance weight matrix was employed (Anselin 1988; Chi and Zhu 2008). Each spatial point in our data has the same exact number of neighbours, but the relative importance (weight) of each neighbour attribute is proportional to its inversed distance (Getis and Aldstadt 2004).

Using this matrix (see Fig. 6 in “Appendix”), a global spatial autocorrelation indicator (the Moran’s Global *I*) was computed for three focal variables.^9^ Because this measure ignores potential instability over space (Anselin 1995), we supplemented our analyses by turning to Local Indicators of Spatial Association (LISA) and specifically to Local Moran’s *I* (Anselin 1995).^10^ In the context of this study, using LISA to identify the spatial clusters and outliers of dependent variables should help us identify the local specificities of family models.

To investigate the extent to which the observed regional patterns result from underlying demographic, socio-economic, or environmental variability, we derive in the third step OLS regression estimates of the associations between the living arrangements of the elderly and a broad range of contextual variables pertaining to our regional populations (see below). Our goal is not to develop formal causal models of the living arrangements of the elderly, but to control for variations in the basic demographic, socio-economic, institutional–cultural, and ecological factors that are likely to affect the residential patterns of older people (cf. Ruggles 2009, 2010; Gruber and Szołtysek 2012).

In the final step, we ask whether the distribution of clusters and outliers identified in step 2 above remains after controlling in the regression for the chosen set of covariates. To answer this question, LISA is applied to the spatial distribution of residuals from the OLS regression models. While the LISA analysis of the models’ residuals is usually considered a regression diagnostic tool (e.g. Anselin 1988, 100 ff), it can also be viewed as a powerful analytical device in itself (e.g. Zolnik 2011; Overman et al. 2009; James and Moeller 2013), as it can provide information on the persistence of spatial clustering after accounting for contextual conditions and further quantification of the spatial structure of the outcome variable.

### Local-Level Control Variables

Before we can draw conclusions about the effects of location-specific societal norms on the residential patterns of the aged, we must be able to prove that these patterns are not attributable to demographic or other factors (Ruggles 1987). Demographers have long known that mortality, fertility, generation length, marriage patterns, and age distribution can set limits on the type and the number of kin available for co-residence, and may therefore affect the capacity of individuals to cohabit with certain types of kin (Wachter et al. 1978; Bongaarts 1983; De Vos and Palloni 1989; Hammel 1990; Smith and Oeppen 1993; Wolf 1994; Hall et al. 1997; Palloni 2001; United Nations 2005; Gaymu et al. 2006; Reher and Requena 2017). In addition to directly influencing residential opportunities in old age, demographic forces may interact with a wide array of socio-economic and cultural factors (Bongaarts 1983).

To account for these potential contextual effects, we selected a set of demographic, ecological, and institutional control variables based on suggestions made in the previous literature (esp. Ruggles 2009, 2010; Gruber and Szołtysek 2012; Szołtysek et al. 2017), albeit with some modifications. Our choice of variables was largely limited to measures that could be computed from our database and that were available for all regional populations of the combined NAPP and Mosaic data (see Table 2).^11^

**Table 2:** Descriptive statistics for dependent and control variables used in the regression models. *Source*: Mosaic/NAPP data. For primary sources of the Mosaic and NAPP data, see Electronic Supplementary Material 1. For technical details on the computation of the geocovariates, see Electronic Supplementary Material 2

	Mean	SD	Median	Min	Max
LMD	0.26	0.15	0.22	0.01	0.74
LLK	0.08	0.12	0.04	0	0.61
LWR	0.2	0.11	0.21	0	0.56
Rural	0.85	0.31	1	0	1
SMAM female	25.21	3.03	26.07	16.76	31.16
SMAM male	27.94	2.43	28.17	19.33	34.33
Married elderly	0.44	0.09	0.43	0.19	0.7
Unmarried women	0.37	0.08	0.37	0.14	0.62
Nonmarriage	0.1	0.07	0.1	0	0.38
Availability ratio	13.5	8.81	11.13	5.54	90.39
Croplands	13.26	10.43	10.63	0	57.43
Population potential	1,205,110	1,035,521	876,850	26	4,607,194
Terrain ruggedness	20.67	27.23	10.57	0.16	217.6
Numeracy (W_*tot*_)	2.6	2.77	1.43	0.16	12.32

Dummy variables for time period, fertility decline, and preference for sons not included

*Male and female marriage age* (SMAMs) are included as important constraints on the frequency of the occurrence of three-generation (extended) families (Ruggles 1987, 63, 191–198), even though their impact on our indicators of co-residence among the elderly may not be unequivocal. Late marriage may shorten the length of time multiple generations overlap and thus can limit opportunities for the aged to co-reside with married descendants. Conversely, in contexts where cultural preferences place a premium on patrilocal multiple-family living, early marriage and long periods of generation overlap may bolster authority structures fostering greater durability of complex residential arrangements (Hammel 1980). A high average age at marriage, especially when it occurs in parallel with the neolocality of married children, can lead to a divergence of the life cycles of the younger and the older generations and may thus decrease the potential for older people to be reincorporated into the households of their offspring (Laslett 1988). On the other hand, people living in societies in which late marriage is common (and fertility control is minimal) may still bear children late in life, thereby decreasing the probability of complete residential isolation in old age (Ruggles 1996b, 24).

*Nonmarriage* (*aka* permanent celibacy; the percentage of persons aged 45–54 who have never married; both sexes combined) directly determines the population at risk of living in multigenerational arrangements (Ruggles 2010). High celibacy rates in a population would also tend to limit the pool of ever-married kin needed to form both the LMD and the LLK arrangements. Generally, high levels of *nonmarriage* are negatively associated with living with relatives and are positively associated with residential isolation in old age.

Our models explicitly include the percentages of elderly men and women who were living with a spouse (*married couples*) and *unmarried* (*and widowed*) *women* (unmarried men are a residual category; Ruggles 2009, 258). The share of older people who are married has a direct impact on the prevalence of primary and secondary unrelated individuals (Ruggles 1988) and is therefore negatively associated with residential isolation among the elderly. At the same time, whereas in most societies demographic realities have meant that elderly women are more exposed than older men to the risk of living alone (Wolf and Soldo 1988), it is also possible that unmarried women are more inclined to fall back on relatives in case of need (Alter 1988, 158–159; Wall 2002), thus making demographic trends less straightforward.

In order to further account for the pool of individuals with whom the elderly could co-reside, a *kin availability ratio* was computed (Palloni 2001; also United Nations 2005, 63) as a ratio of the population aged 15–64 (an age range that includes most of the children and some of the younger siblings of the older population) to the population aged 65 and older across the populations studied.^12^ Research on contemporary developing countries has shown that for elderly people, kin availability is positively related to the probability of living with children or other relatives and is negatively related to the probability of residential isolation from kin (United Nations 2005, 64–65).

Finally, we included an indirect measure of the onset of a monotonic fertility decline (dummy) that we derived by matching our regional–temporal data with province-level estimates of the onset of the fertility decline from the Princeton European Fertility Project’s capstone volume (Coale and Watkins 1986).^13^ We hypothesised that in regional populations with declining fertility, there would be fewer children with whom the aged could co-reside, but that the availability of lateral kin would be largely unaffected (Ruggles 1996a, b).

We also controlled for urban–rural distinctions across our data. In historic contexts, living in an urban area may signify exposure to higher mobility and migration levels, which could limit the number of kin available for co-residence. Moreover, due to sex-selective migration patterns, marriage markets were often skewed in urban populations. This means that large fractions of women remained unmarried and were thus prone to residential isolation in old age (Kok and Mandemakers 2015; cf. Martin and Kinsella 1994).

We use the *population potential* covariate (see Stewart and Warntz 1958) to account for whether a region was more centrally or more peripherally located. Given the opportunity structures related to mobility, wage labour, and employment (affecting both younger and older generations), we expect to observe lower levels co-residence with kin among elderly in areas close to important population centres than in sparsely populated regions, where relatives might share a household as a form of protection against socio-economic or environmental vicissitudes (Palloni et al. 2009). In addition, large numbers of people living in close proximity are more likely to be able to maintain kinship ties without living together (Enke 2018). Overall, we expect to observe a positive association between population potential and living without kin and a negative association between population potential and stem- and joint-family arrangements.

Living in an area with rugged terrain is another factor that could foster residential crowding and limit the risk of residential isolation (Szołtysek 2015). Rugged topography frequently represents an obstacle or barrier to meeting, communicating, and interacting (see Jimenez-Ayora and Ulubaşoğlu 2015), and these constraints may lead to a preference for collectivist modes of social behaviour and for co-residence with kin in old age (Szołtysek et al. 2017). The variable *terrain ruggedness* (Wilson et al. 2007) is used to control for these effects.

Given that the extent of elderly familial isolation could be a function of a region’s institutional and economic development (e.g. United Nations, 2005, ch. III), regional estimates of *numeracy* derived from measures of age-heaping in each regional data set were included in the model (whereby a lower prevalence of age-heaping implies a higher level of numeracy). Numeracy has been used extensively in the economic history literature as a proxy for human capital levels in historic populations (Tollnek and Baten 2016) and as a broad indicator of institutional modernisation (A’Hearn et al. 2016). Based on this literature, we hypothesise that in regions with higher levels of age-heaping (i.e. lower levels of numeracy), the proportions of the aged who were living without any relatives were lower.^14^

In addition, information on the historical share of croplands has been used as a crude proxy for the role of agriculture. The positive effect of farming on intergenerational co-residence has been stressed by a long line of scholars (e.g. Ruggles 2009).

Cultural differences in the desirability of intergenerational contact (i.e. “normative solidarity”) are also likely to induce variation across regions in patterns of co-residence among the elderly (Reher 1998; Palloni 2001, 86–88), but these preferences are particularly difficult to measure for past societies. As an indirect measure of such attitudes we used the *sons’ preference* index developed by Gruber and Szołtysek (2016).^15^ Given that a wide range of cross-cultural research has found that the preference for sons is a good indicator of intergenerational solidarity (e.g. Das Gupta et al. 2003; also Szołtysek et al. 2017), we expect to find that this variable was positively related to living with relatives and was negatively related to living alone in old age.

We also controlled for the period in which all or most of the data for each of the regional populations were collected, distinguishing between the following periods: pre-1800, 1800–1850, and after 1850 (reference).

Finally, to explore the degree to which each of our models is affected by spatial autocorrelation, we derived the Moran’s *I* index of spatial autocorrelation for the model residuals based on the spatial weights matrix specified above. If the model’s residuals show significant spatial autocorrelation, the OLS assumptions about the independence of the observations might be violated, which could bias the coefficient estimates.

## Results

### Unconditional Mapping Analysis

**Fig. 1:**
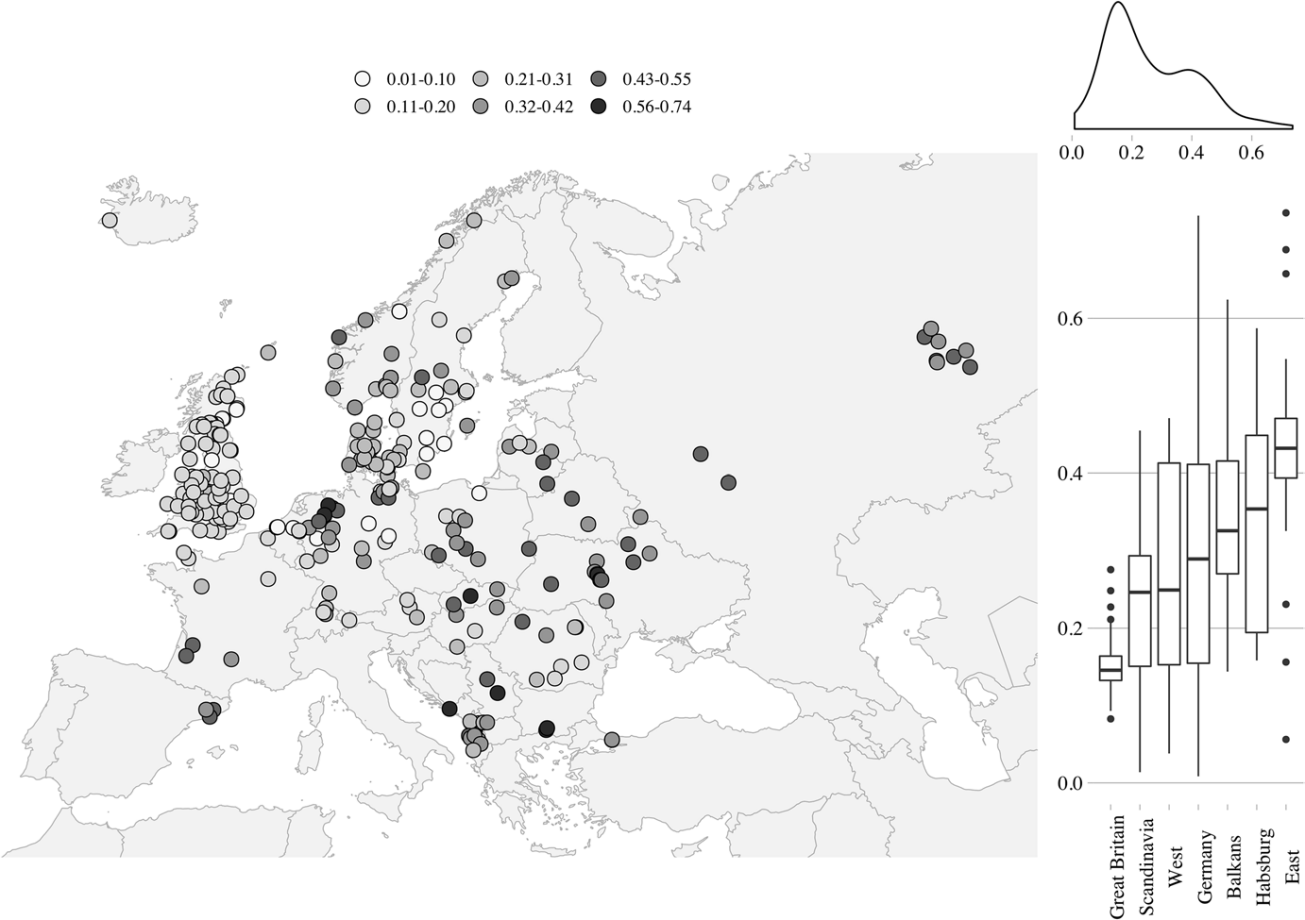
Statistical and spatial distribution of the proportion elderly living with one ever-married descendant (LMD). *Notes*: each point on the map represents one Mosaic/NAPP regional population as defined in the text. Seven bigger territorial groupings on the right-side panel of the figure followed major institutional and socio-economic distinctions across historic Europe. “Great Britain”: England, Wales, and Scotland; “Scandinavia”: Danish, Swedish, and Norwegian data, as well as Iceland; “Germany”: German-dominated areas other than the Habsburg territories; “West”: areas west and south-west of Germany; “Habsburg”: Austrian, Hungarian, Croatian, as well as Slovakian data; “East”: east-central and Eastern Europe, including the former Polish-Lithuanian Commonwealth and Russia; “Balkans”: areas south and/or east of Croatia and Hungary. *Source*: Mosaic/NAPP data. For primary sources of the Mosaic and NAPP data, see Electronic Supplementary Material 1

**Fig. 2:**
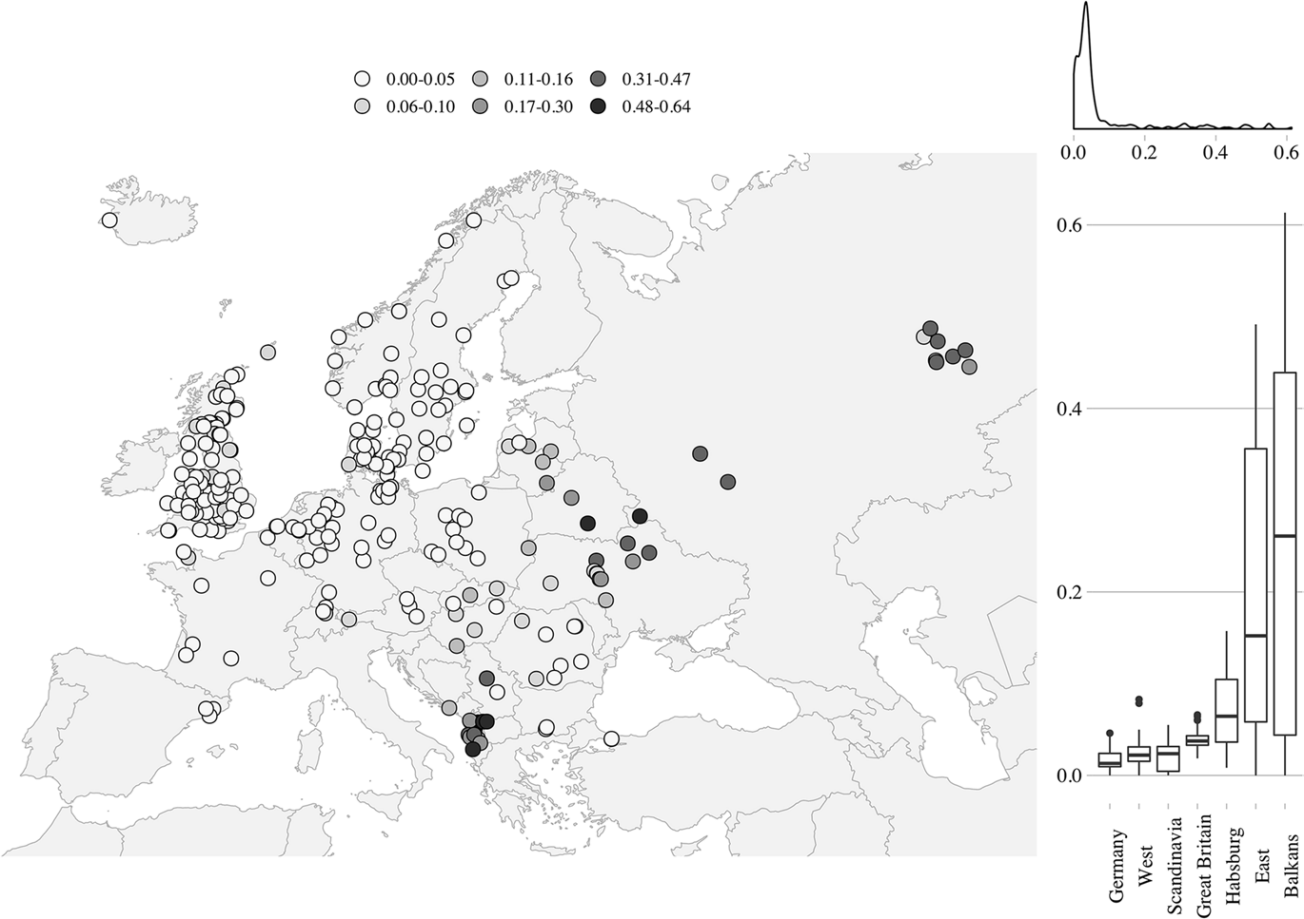
Statistical and spatial distribution of the proportion elderly living with 2+ ever-married descendants or ever-married lateral kin (LLK). *Note*: each point on the map represents one Mosaic/NAPP regional population as defined in the text. Seven bigger territorial groupings on the right-side panel of the figure defined as in Fig. 1. *Source*: Mosaic/NAPP data. For primary sources of the Mosaic and NAPP data, see Electronic Supplementary Material 1

**Fig. 3:**
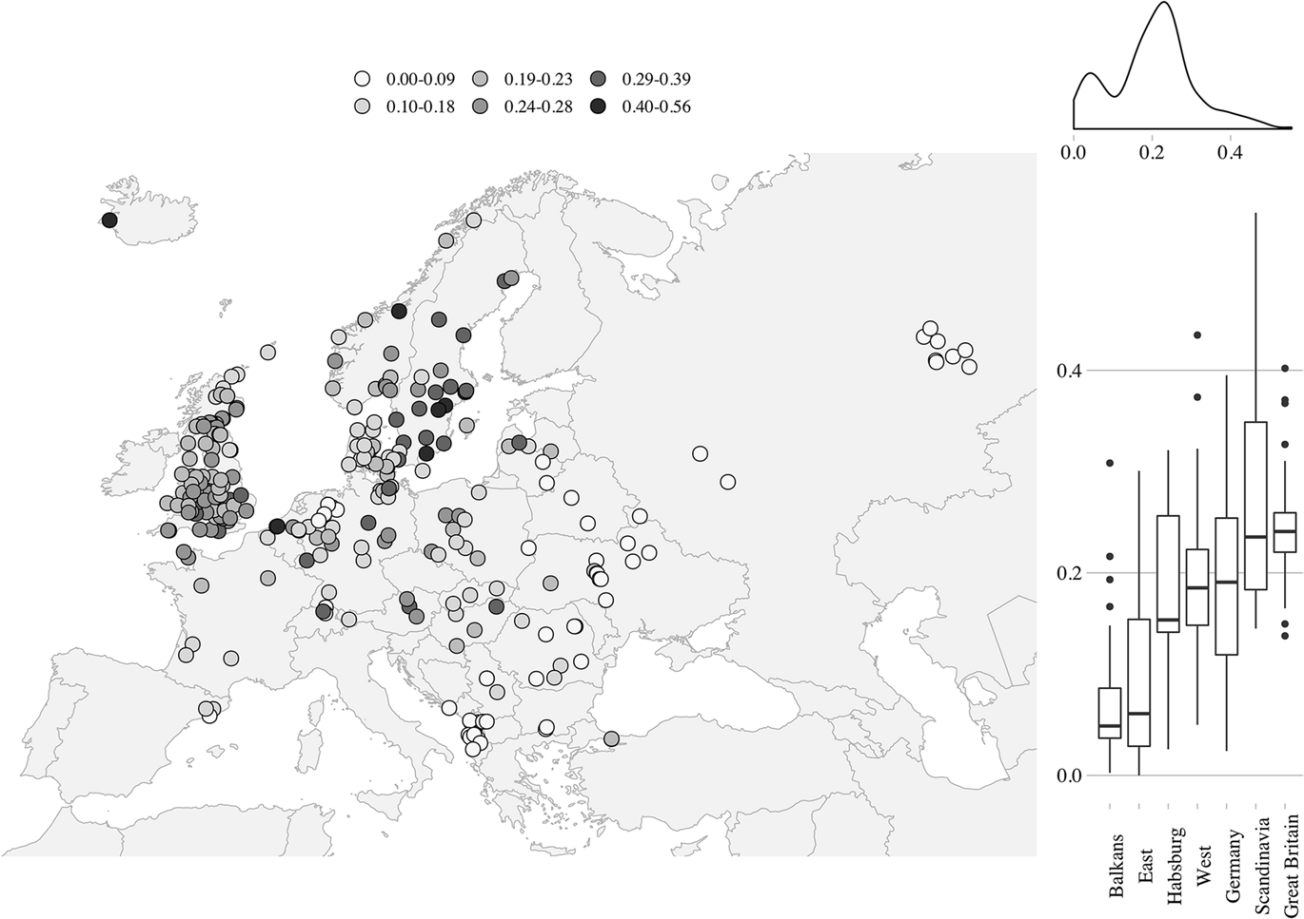
Statistical and spatial distribution of the proportion elderly living without any relatives (LWR). *Note*: each point on the map represents one Mosaic/NAPP regional population as defined in the text. Seven bigger territorial groupings on the right-side panel of the figure defined as in Fig. 1. *Source*: Mosaic/NAPP data. For primary sources of the Mosaic and NAPP data, see Electronic Supplementary Material 1

Our results start by considering the statistical and spatial distribution of our focal variables (Figs. 1, 2, 3).

According to Fig. 1, the majority of regions under analysis contained a non-negligible fraction of the aged living in stem-family-like arrangements (the grand mean = 26.1%; median = 21.8%). Some areas appear to have a high degree of homogeneity (e.g. very low values in southern Sweden or medium values in England, Wales, and Scotland), while others display a high degree of heterogeneity (particularly Germany). Overall, however, the spatial distribution of the variable is far from random. In terms of global autocorrelation, Moran’s *I* of 0.74 (*p *< .001) indicates that there are high and significant tendencies for regions or sets of regions with similar values of this variable to cluster close to each other.^16^ The LMD values become somewhat more generalised in the east, where, on average, more than four in 10 elderly people were living with one married descendant. However, comparable proportions of the aged living in such arrangements are found in various locations in the Habsburg and the western territories and in the German lands in particular.^17^ Moreover, the higher LMD values do not seem to form a clear geographic pattern. Medium values of elderly people living with one married descendant are dispersed over the entire territory covered by the data set, except for Great Britain. Regions with high and very high values of the variable are even more spread out, stretching over Catalonia, north-western Germany, Slovakia, Serbia, Bulgaria, and central Ukraine. The position of England (in 1881) stands out, as this location has the lowest median values for this variable and much less heterogeneity than any other region.^18^

Figure 2 suggests there is a strong spatial autocorrelation at the global level for our second variable as well. The coincidence of value similarity with locational similarity that seems evident from a graphic visualisation is confirmed by the strongly positive and significant Moran’s *I* of 0.81 (*p* < .001).^19^ The map reveals two modes of familial behaviour with respect to the elderly: a complete aversion to the joint-family-like configurations in much of western, central, and Nordic Europe; and a strong preference for this co-residential pattern in the east and south-east. It is especially noteworthy that the lowest values are observed not in the north-western European “core” areas, but in Scandinavia and Germany. Similarly, low values of the LLK variable are also found in Poland and Romania. Again, the degree of variation within regions can be very high. The Balkan territories are particularly heterogeneous, as joint-family-like configurations are widespread in some areas, while low “western-like” values are dominant in others, particularly in the Romanian territories, some parts of Bulgaria, and Istanbul city. These findings suggest that a straightforward east–west distinction might be difficult to sustain in our analysis (cf. Hajnal 1983), even though it is clear that regions ranging from Russia, Belarus, Ukraine, and Albania diverge from the rest of the data set, as both forms of intergenerational co-residence (LMD and LLK) are observed in these territories.

A similar pattern emerges for the regional distribution of the LWR (Fig. 3), which is reflected in the high and positive value of Moran’s *I* (0.69; *p *< .001).^20^ Populations with values under 10% (indicating that very low shares of elderly people were living without kin) are almost exclusively located in south-eastern and Eastern Europe. However, Westphalia again differs from the rest of Western Europe, yielding values very close to those observed in the east. The highest percentages of elderly people who were not living with relatives are found in Scandinavia and the Netherlands. Whereas medium values are observed across Great Britain, the values detected in Scandinavia are more diverse. The Swedish census of 1880 in particular has clusters of extremely high values, which may represent historical antecedents of the current tendency in Sweden to live alone (Kohli et al. 2005; Hank 2007). German territories are also very diverse, including data points at both ends of the scale. The broad terrain of east-central Europe appears split into its western-central and eastern parts, apparently along the famous “Hajnal line” (Hajnal 1983).

### Local Spatial Regimes

**Fig. 4:**
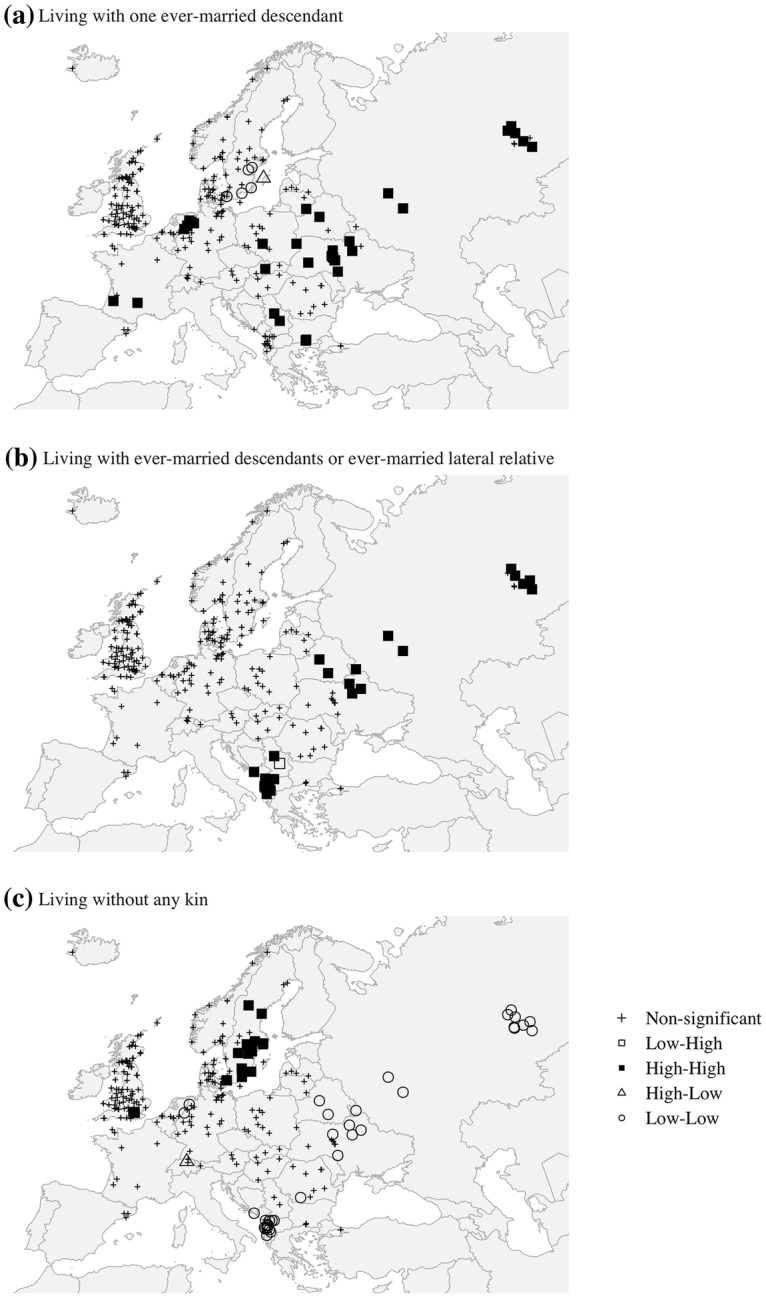
LISA significant clusters for the distribution of measures of elderly living arrangements. *Note*: each point on the map represents one Mosaic/NAPP regional population as defined in the text. *Source*: Mosaic/NAPP data. For primary sources of the Mosaic and NAPP data: see Electronic Supplementary Material 1

Having established a nonrandom structure in the global spatial distribution of our variables, it is instructive to explore how those clustering trends could be understood in terms of local geographic patterns. In order to appreciate the regional structure of spatial autocorrelation, the next set of figures (Fig. 4) presents the local Moran’s *I* for our three focal variables, respectively. Spatial clusters are identified for the locations where the values of a particular variable are more similar to those of its neighbours than they would be if they were randomly distributed.^21^ The null hypothesis is that the values being analysed exhibit a random spatial pattern, and the LISA clusters are marked when their values are significant at least at the 95% level, indicating the regions that make the most meaningful contributions to the global autocorrelation outcome.

Figure 4a strengthens the results for the previously obtained LMD. The local pattern reflects the global trend towards positive spatial autocorrelation: all of the regions (with the sole exception of Gotland) tend to cluster based on the similarities rather than the differences between their attribute values, and 85% of the significant local clusters fall into the *high*–*high* category. Within this global structure, we can identify several significant local regimes of high LMD values that stretch from southern France; through north-western Germany, southern Poland, and Slovakia; reaching western Ukraine and European Russia in the east; and extending further towards the central Balkans. There is evidence also of the *low*–*low* type regime in southern Sweden, where the strong aversion to stem-family-like formations has already been noted. The lack of hot spots in much of Great Britain confirms our earlier observation that the region displays similarly moderate values across its territory.

The local clustering of elderly people living with lateral kin corroborates our previous finding that there is a considerable lack of balance in the spatial distribution of the data (Fig. 4b). All of the clusters with a significant positive association are in the *high*–*high* category, and all are located in the south-eastern and eastern parts of the studied area. This evidence of spatial dependency therefore confirms our finding that the Belarusian, northern Ukrainian, Russian, and Albanian family arrangements contribute most to the global spatial autocorrelation outcome. Notably, previous findings of pockets of higher values in several other Eastern European areas, such as in Latvia, Poland, western Ukraine, and Hungary, are not confirmed. On the other hand, the failure to detect significant *low*–*low* clusters may be misleading given the highly skewed distribution of the data and the spatial proximity of extreme values.

Figure 4c also confirms our earlier exploration of the raw data. With 98% of the significant local clusters falling into the *high*–*high* or *low*–*low* categories, it reiterates the global trend towards positive spatial autocorrelation we noted before. Unlike the previous variable, the LWR identifies two distinct spatial clusters of high and low values. This finding of spatial heterogeneity mainly reflects the distinction between the northern locations on the one hand and the eastern and the south-eastern locations on the other. Most of the cold spots are located in the Balkans and in the Eastern European regions, which indicates that there is a strong local clustering of regions or groups of regions where the aged were almost always living with relatives, while Sweden displays the opposite pattern. A *low*–*low* cluster of regions found in the Westphalia region of Germany challenges the assumption that there is a straightforward east–west dichotomy in the LWR. The city of Zurich appears to be the only *high*–*low* spatial outlier. Our failure to detect a clear local spatial structuring of the variable over much of eastern-central and Western Europe, or across England and Wales and the Danish–Norwegian territories, suggests that the patterns in these regions are not significantly different from random.

### Multivariate Regression Results

In order to check how sensitive the findings presented above are to the contextual effects that could influence the residence patterns of the elderly shown in our data, we specify for each of our focal measures a multiple linear regression model using ordinary least squares (OLS) with the control variables listed above. To control for the overrepresentation in our sample of populations from certain parts of Europe (e.g. Great Britain or Scandinavia) and the underrepresentation of populations from others (e.g. Western Europe), we decided to apply weights to ensure that each of the seven bigger territorial groupings (defined as in Fig. 1) is given equal weight in the regression models.

Since the outcomes for particular independent variables are not the immediate focus of our analysis, we discuss them only briefly (Table 3).^22^ The control variables account for a non-negligible part of the variation in our measures of co-residence among the elderly in every model.^23^ The significant influence of the census time period and the timing of the population’s fertility transition can be observed for the first variable only. Moreover, only some of the demographic predictors are shown to attain significance in every OLS model. The proportions of elderly people who were married men or women, unmarried women, or never-married men or women are all negatively correlated with the shares of elderly people living in the two distinct forms of complex family arrangements, but are positively correlated with the proportion of the elderly living without relatives—which is plausible. The only other variable for which the effect is found to be equally consistent is our measure of institutional modernisation (age-heaping/numeracy). A high number of people misreporting their age in each region (lower numeracy) are shown to be associated with, on the one hand, a diminishing proportion of the elderly living without any kin; and on the other hand, increasing values of the two measures of complex co-residence patterns—which is in line with our expectations.

**Table 3:** OLS regressions of contextual characteristics on the living arrangements of the aged. *Source*: Mosaic/NAPP data. For primary sources of the Mosaic and NAPP data: see Electronic Supplementary Material 1. For technical details on the computation of the geocovariates, see Electronic Supplementary Material 2

	LMD			LLK			LWR		
	β	SE	*p*	β	SE	*p*	β	SE	*p*
Rural	− 0.03	0.04	0.506	0.09	0.04	0.041	0.02	0.04	0.595
1800–1850	− 0.29	0.07	< .001	− 0.12	0.06	0.060	0.10	0.06	0.090
After 1850	− 0.21	0.08	0.012	− 0.06	0.08	0.434	0.09	0.07	0.230
SMAM female	0.01	0.10	0.906	− 0.51	0.10	< .001	0.11	0.09	0.190
SMAM male	0.09	0.08	0.266	0.03	0.07	0.653	− 0.10	0.06	0.130
Married elderly	− 0.43	0.10	< .001	− 0.56	0.10	< .001	0.30	0.09	< .001
Unmarried women	− 0.18	0.09	0.043	− 0.23	0.09	0.009	0.38	0.08	< .001
Nonmarriage	− 0.61	0.07	< .001	− 0.16	0.07	0.015	0.29	0.06	< .001
Availability ratio (ln)	0.07	0.06	0.256	− 0.23	0.06	< .001	0.10	0.05	0.067
After fertility transition	0.10	0.05	0.046	− 0.05	0.05	0.278	0.05	0.04	0.179
Croplands	− 0.04	0.05	0.407	− 0.06	0.05	0.211	0.02	0.04	0.570
Population potential (ln)	− 0.07	0.06	0.261	− 0.22	0.06	< .001	0.02	0.05	0.748
Terrain ruggedness (ln)	0.09	0.05	0.071	0.02	0.05	0.600	− 0.13	0.04	0.001
Son preference 1–3	0.05	0.05	0.310	0.04	0.05	0.455	− 0.03	0.04	0.434
Son preference > 3	− 0.09	0.05	0.038	0.09	0.04	0.037	0.01	0.04	0.843
Numeracy (W_*tot*_ (ln))	0.26	0.08	0.002	0.26	0.08	< .001	− 0.51	0.07	< .001
Intercept	Yes			Yes			Yes		
N	277			277			277		
*R*^2^/adj. *R*^2^	.513/.483			.605/.581			.629/.607		
F-statistics	17.112***			24.939***			27.591***		
AIC	− 356.608			− 495.112			− 643.233		
Moran’s *I*	0.458***			0.406***			0.256***		

***p < .001

The significance of the other control variables is found to be less consistent across the models. The co-residence with lateral kin variable is shown to be associated with the decrease in female SMAM and the availability ratio. The former result is plausible given that a low female age at marriage reduces the age differences between generations and increases the potential for intergenerational co-residence. At the same time, a lower population potential and a higher level of rurality (both of which indicate a lower level of urbanisation) are associated with an increasing proportion of the elderly were living with lateral kin. Thus, these outcomes corroborate previous results suggesting that the more diversified social structures of cities and densely populated regions were less conducive to the formation of very complex family structures (Szołtysek et al. 2017; Ruggles 2009). The ruggedness of the terrain is found to be significant only in the case of the LWR variable (negative effect), which suggests that elderly people were less likely to live alone if they had opportunities to communicate with individuals living nearby—which also makes sense. The highest level of son preference is found to be positively correlated with an increase in the values of the LLK variable and with a decrease in the likelihood of living with a married descendant. Although the latter observation is difficult to interpret, the former finding is in line with the well-established positive association between gender asymmetries and the prevalence of “joint-family-like” structures (Szołtysek et al. 2017).

Although the outcomes of the OLS models described above are highly significant and explain 48% or more of the variation in the dependent variables, they are also affected by the strongly positive spatial autocorrelation. In each case, the Moran’s *I* test on the models’ residuals is shown to be significant at the 0.001 level. This suggests that contrary to the OLS assumptions, the observations are not independent, which could bias the coefficient estimates. This finding prompted us to check the robustness of the OLS results by computing spatial error models. Unlike the standard OLS models, this type of regression assumes a spatial correlation of errors. For spatially autocorrelated OLS models, it can be used to correct the impact of spatial correlation and avoid the overestimation of the impact of independent variables on the dependent variable (Ward and Gleditsch 2008). Although the new estimates are not identical with the OLS results described above (see Table 4 in “Appendix”), the differences are very small and reflect changes in the significance of only some of the predictors. No changes in the direction of the estimates are detected. These results indicate that despite the presence of spatial autocorrelation, the general outcomes of the OLS models should not be discarded as flawed.

**Table 4:** Results of the spatial error models. *Source*: Mosaic/NAPP data. For primary sources of the Mosaic and NAPP data: see Electronic Supplementary Material 1. For technical details on the computation of the geocovariates, see Electronic Supplementary Material 2

	LMD			LLK			LWR		
	β	SE	*p*	β	SE	*p*	β	SE	*p*
Rural	0.039	0.033	0.229	0.140	0.030	< .001	− 0.050	0.032	0.121
1800–1850	− 0.326	0.068	< .001	0.052	0.064	0.413	0.029	0.062	0.640
After 1850	− 0.198	0.077	0.010	0.184	0.071	0.010	0.009	0.072	0.900
SMAM female	0.147	0.094	0.118	− 0.320	0.088	< .001	0.136	0.087	0.120
SMAM male	− 0.019	0.071	0.786	− 0.258	0.066	< .001	− 0.103	0.066	0.116
Married elderly	− 0.320	0.091	0.000	− 0.178	0.085	0.035	0.181	0.086	0.036
Unmarried women	− 0.050	0.083	0.549	− 0.142	0.077	0.065	0.331	0.077	< .001
Nonmarriage	− 0.473	0.071	0.000	0.052	0.066	0.431	0.309	0.065	< .001
Availability ratio (ln)	0.049	0.055	0.372	− 0.141	0.051	0.005	0.055	0.052	0.286
After fertility transition	− 0.037	0.043	0.396	− 0.051	0.040	0.199	0.091	0.041	0.026
Croplands	0.031	0.041	0.451	− 0.023	0.038	0.546	0.016	0.039	0.686
Population potential (ln)	− 0.048	0.078	0.538	− 0.212	0.076	0.005	0.041	0.063	0.515
Terrain ruggedness (ln)	0.193	0.052	< .001	0.085	0.049	0.083	− 0.144	0.045	0.002
Son preference 1–3	0.018	0.036	0.609	0.042	0.033	0.204	− 0.022	0.035	0.531
Son preference > 3	− 0.112	0.034	0.001	0.045	0.032	0.152	0.031	0.033	0.355
Numeracy (W_*tot*_ (ln))	0.160	0.080	0.046	0.107	0.075	0.156	− 0.325	0.073	< .001
Lambda	0.628	0.040	< .001	0.665	0.036	< .001	0.479	0.052	< .001
Wald statistics	243.8***			325.65***			84.585***		
Intercept	Yes			Yes			Yes		
N	277			277			277		
Log likelihood	− 281.9828			− 263.2436			− 262.8105		
AIC	601.97			564.49			563.62		

***p < .001

### LISA on Model Residuals

**Fig. 5:**
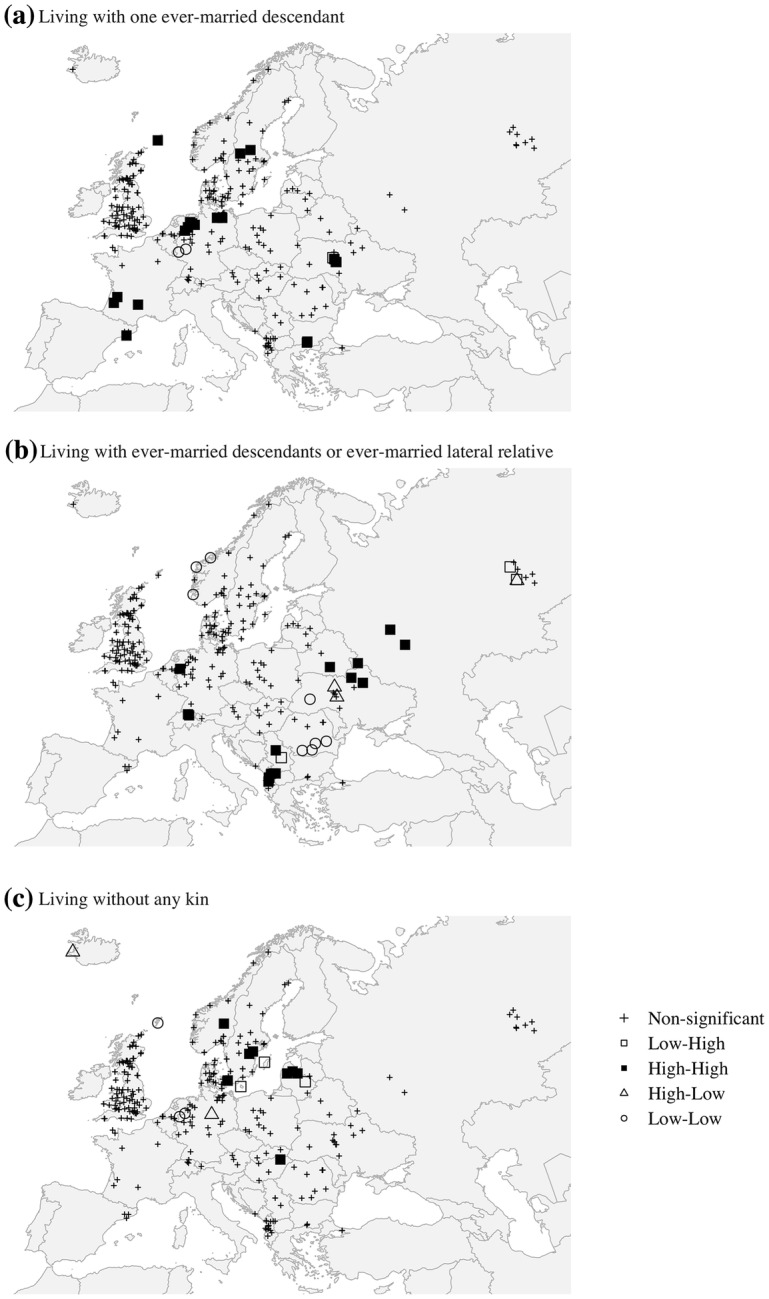
LISA on the residuals of the regression predicting the living arrangements of the aged. *Note*: each point on the map represents one Mosaic/NAPP regional population as defined in the text. *Source*: Mosaic/NAPP data. For primary sources of the Mosaic and NAPP data: see Electronic Supplementary Material 1

**Fig. 6:**
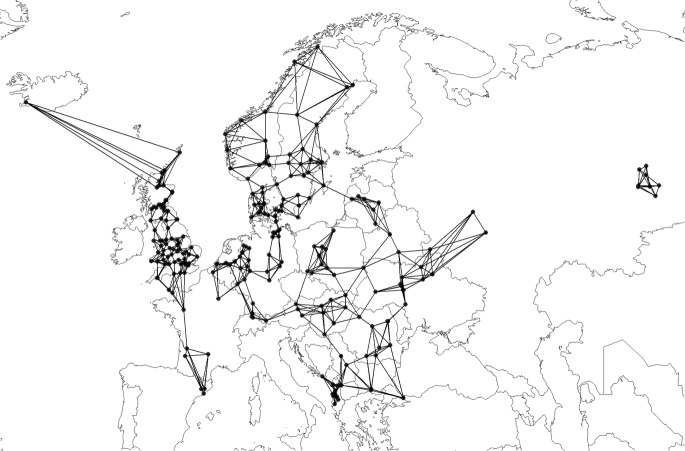
Connectivity matrix of the NAPP/Mosaic data. *Note*: each point on the map represents one Mosaic/NAPP regional population as defined in the text. The five-nearest neighbours network structure (based on the great-circle distances) with row-standardised inverse distance weight matrix was employed. Each spatial point in our data has the same exact number of neighbours, but the relative importance (weight) of each neighbour attribute is proportional to its inverse distance. *Source*: Mosaic/NAPP data. For primary sources of the Mosaic and NAPP data, see Electronic Supplementary Material 1

To determine whether local spatial clustering persists after controlling for demographic, socio-economic, and environmental covariates in the regressions, LISA analysis was performed on the OLS models’ residuals (Fig. 5). These results should thus be compared with those presented earlier (Fig. 4). In the figures, the *high*–*high* clusters now indicate regions where the regression model underestimated the outcome variable (i.e. the observed values are higher than predicted), while the *low*–*low* clusters point to regions where the model overestimated the outcome variable (i.e. the observed values are lower than predicted).

When looking at Fig. 5a, it is worth noting that although the control variables filter the spatial association in the LMD variable to some extent (Moran’s Global *I* on model residuals equals 0.46 with *p* < .0001, compared to 0.74 for the raw data), the association remains high at both the global and the local level. Although highly influential leverage points that drive the global measure can still be detected, their geographic distribution is less concentrated and their pattern is less clear than in the previous analysis of the raw data. Notably, we find that a number of regional clusters that are most evident in Fig. 4a have been filtered out by the model: namely, the *low*–*low* clusters in southern Sweden and the *high*–*high* pockets in central and Eastern Europe and in the Balkans. This outcome may indicate that part of the previously observed spatial nonstationarity in these regions resulted from a broad mix of contextual factors that are controlled for in the regressions. On the other hand, the regression model still fails to reduce the local spatial autocorrelation found in regions such as Westphalia, south-western France, parts of Ukraine, and southern Bulgaria. The strong presence of LMD traits that is detected in those regions—and that is much stronger than would be expected on the basis of the demographic, socio-economic, and environmental characteristics of these populations—can be attributed to some unobserved characteristics of these places or, alternatively, to the local specificity of the prevalent family model.

Similar procedures are repeated for the LLK variable (Fig. 5b). Although the global spatial dependence shrinks (Moran’s Global *I* decreased from 0.81 to 0.41; both values highly significant) and the number of *high*–*high* clusters is reduced when local spatial autocorrelation is assessed for model residuals, the spatial patterns do not disappear. Some previously identified hot spots of high proportions of elderly people living with lateral kin are filtered out by our control variables, especially in parts of Albania and in the Urals. However, spatial conglomerates in present-day Belarus, northern Ukraine, and the western part of European Russia remain unaltered. In these areas, the observed values of the LLK are significantly higher than would be expected given the demographic, socio-economic, environmental, and institutional characteristics of these populations. The same pattern is found for the spatial outlier in southern Serbia. The effort to explain the clusters of high values results in the significant overestimation of the response variable in the regions where its values are low (e.g. in the “Bible Belt” are of Norway and in southern Romania).

Finally, Fig. 5c displays the results of the assessment of the spatial autocorrelation for residuals of the model predicting the regional shares of elderly people living without relatives. Compared to the results shown in Fig. 4c, the application of this procedure reduces the global spatial autocorrelation (Moran’s Global *I* changes from 0.68 to 0.26). At the same time, this exercise helps explain several significant local data conglomerates that were previously identified. In particular, quite a number of hot spots in Scandinavia and all of the cold spots in the Balkans and Eastern Europe are wiped out by controlling for contextual factors. However, the application of the LISA procedure does not change the significant *low*–*low* type clusters found in north-western Germany, where the observed values were lower than predicted. Again, we find that the resulting spatial distribution of significant clusters is not geographically straightforward.

## Discussion and Conclusions

In this paper, we illustrated the benefits of using an explicitly spatial data analysis to examine a number of longstanding puzzles of historical demography, including the question of whether the varying distribution of living arrangements among the elderly reflects significant differences between major areas of Europe and the question of how sensitive that observed pattern is to the effects of potential determinants of residential choices. Does the picture our data have allowed us to draw confirm the conventional assumption that there was an east–west dichotomy in familial behaviour, or does it suggest that there were spatiostructural complexities that indicate more nuanced geographies of residence patterns among the elderly?

Our data set, exploratory spatial data approach, and efforts to control for a number of potentially confounding factors have provided us with a comprehensive data and methodology framework to engage with these issues. First, by combining cross-sectional census and census-like data from the Mosaic and NAPP projects, we were able to generate the first nearly pan-European picture of historic co-residence patterns among the elderly. Second, by applying the ESDA tools, which are still relatively new to historical demography, we were able to examine explicitly the historical geography of the living arrangements of the elderly and thus to provide a picture that is more nuanced than the conventional portrayals of historical family patterns in Europe. Third, by explicitly allowing our populations to differ in their contextual characteristics in the regression framework, we have responded to numerous pleas made in the literature (e.g. Ruggles 2009, 2010, 2012) that controlling for demographic, socio-economic, and institutional conditions is essential when comparing living arrangements between populations.

Our findings reveal that the historical living arrangements of the European elderly were indeed spatially structured. Using data on 277 European regions, we found strong evidence of global and local positive autocorrelation in the living arrangements of the elderly. Our results indicate that regions where the population had relatively strong (or weak) tendencies to live in specific familial configurations were more frequently localised close to other regions with relatively high (or low) values of the respective variables than when this localisation was purely random. Deviations from that global trend across the three variables (i.e. a negative form of spatial association) were shown to be marginal. It thus appears that at the global level, the spatial order in historic Europe was characterised by more similarities than differences.

Next to global and local dependence, the application of LISA uncovered a substantial regional heterogeneity. Our finding that there were *hot spots* and *cold spots* in the spatial distribution of our focal variables (sometimes simultaneously) suggests that there were large spatial disparities between European regions with regard to the living arrangements of the elderly, and thus confirms family historians’ early assertions that the extremes of familial organisation were present in Europe (Laslett 1977). However, while we indeed detected substantial variation in co-residential arrangements among the aged—i.e. in the extent to which older people were residentially integrated with or isolated from family collectives—the spatial structures we identified do not fully conform to the geographic patterns predicted by the earlier literature, and in many instances, they appear to be strikingly different from the expected patterns.

Overall, our findings challenge a number of “master narratives” on the European geography of family forms advanced by Le Play, Hajnal, Laslett, and their followers (e.g. Todd). The moderate levels of familial complexity among the aged (LMD) that we observed did not appear in one particular area of Europe only. Our results suggest that the existing “map” of the spatial distribution of stem families on the continent (Fauve-Chamoux and Ochiai 2009; Todd 2011) needs to be amended to accommodate central and Eastern European territories for which the occurrence of the LMD arrangements has been documented. Furthermore, while the position of Great Britain was indeed found to be exceptional (at least in 1881), the population in this country was distinguished primarily by its high degree of uniformity, rather than by the presumed “nullity” of stem-family-like arrangements (Laslett 1970, 76–77). The results regarding the most complex family arrangements (LLK) appear to confirm earlier historical demography assumptions that these family forms were strongly clustered in the east (also Ruggles 2010; Gruber and Szołtysek 2012). However, while the findings on the residential isolation of the aged (LWR) seem to be closely aligned along the east–west axis, they are complicated by the presence of significant outliers in the west and especially by hot spots of strong familialistic patterns in Westphalia.

Indeed, one specific gravamen of this paper is that we were unable to find a single territory that could be categorised as “Eastern Europe” with regard to the co-residence patterns of the elderly. Without denying that the east had some peculiarities, the claim that there was a demographically uniform population from the Oder to the Urals cannot be sustained. The residential arrangements of the aged were found to differ from those considered typical of the east in the Polish, Bohemian, and Romanian territories. Moreover, the family patterns we observed in Belarus, Latvia, Lithuania, and Ukraine were not the same as those identified in Russia (cf. Todd 1985). Unfortunately, we were unable to fully explore the north–south dimension of variation across Europe (Reher 1998). Nevertheless, the substantial variability in the living arrangements of the elderly that we found for the northern (Scandinavian) regions in our data set is a warning to avoid making hasty generalisations about the historical experiences of this vast European terrain (cf. Moring 2016).

A substantial share of the global variation observed across our data seems to be driven by demographic, environmental, socio-economic, and institutional variability. For example, the majority of hot spots in the LMD and LWR patterns in the east, as well as the cold spots in these patterns in Scandinavia, appear to be linked to this underlying variability. Nonetheless, a large share of regional specificity in the co-residence patterns of the elderly was found to persist even after these various factors were controlled for. This finding may indicate that in some of the areas we studied, the living arrangements of the aged were determined less by demography and other contextual factors and more by the resilience of the local specificities of family models. Further research should strive to explore more directly the cultural and historical underpinnings of family organisation in these areas.

Finally, a number of limitations of this study must be acknowledged. In this article, the contextual factors were considered only for the purpose of isolating their potential effects on the residential behaviour of the aged, but these factors might also be studied more directly. For example, researchers may want to explore the question of whether the observed spatial inequality in the distribution of the models’ residuals is attributable to an agglomeration of local stories with no common denominator or a macro-level process with local deviations. A future analysis could explore these spatial contingencies more directly by seeking to identify spatially varying associations between that factors that underlie the co-residence patterns of the elderly. Exploring the possible variability of regression coefficients in geographic space could help us determine whether a uniform model of family forms among the elderly is indeed obtainable. The application of more advanced spatial econometric models (e.g. geographically weighted regression) could help to advance the research presented in this paper.

Regarding the data, we must admit that important areas of Europe are not yet covered by the Mosaic/NAPP database, and that those that are covered are sometimes represented by spatially sparse data points. However, given that the processes used to generate the global picture were spatially strongly autocorrelative—and given that Tobler’s “First Law of Geography” (Tobler 1970) states that locations that are close to each other are likely to be similar—we can assume that adding a few data points between the existing locations would not alter the general thrust of our results.

As our analyses were, by necessity, limited to indicators of the position of elderly people based on their co-residence patterns within the household, they pertain only to observable household patterns and not to broader patterns of elderly sociability. In the future, this research could be greatly advanced through the use of social network data that provide information on the larger (i.e. beyond the household) networks of the elderly (see Mönkediek and Bras 2014). Data from large-scale historical databases based on population registers could serve this purpose.

### Electronic supplementary material

Below is the link to the electronic supplementary material.

Supplementary material 1 (PDF 668 kb)

## Appendix

See Fig. 6 and Table 4.

## Footnotes

1 Throughout the paper, living arrangements refer to household co-residential groups and thus need to be distinguished from the residential proximity of separate housing units. Accordingly, the relatives of the elderly are defined as co-resident individuals who are related to the older person by blood or marriage or who have kinship ties established through adoption.

2 See www.censusmosaic.org; https://www.nappdata.org/napp/.

3 In choosing the NAPP data, we gave preference to the oldest available censuses for Iceland, Denmark, and Norway (eighteenth to early nineteenth centuries), while the earliest NAPP data for Sweden come from the late nineteenth century (1880). We use the 1881 census of Great Britain rather than the 1851 census that was recently made available, because the latter is less complete than the 1881 census and has some ambiguity regarding the coding of boarders and lodgers (see Schürer et al. 2019). Except for England, where we employ a 10-per cent sample, we use 100-per cent samples. All other data from Great Britain represent 100-per cent samples.

4 As a rule of thumb, we ensured that each Mosaic region had at least 2000 inhabitants and that urban and rural settlements were separated.

5 In general, the Mosaic data consist of regions formed by one or more locations that were either urban or rural, while the NAPP data were analysed according to the regional division in the census. This implies that regions based on the NAPP data usually comprise both urban and rural populations. The definition of urban was not the same across all the NAPP data, but we took the information provided in the microdata. As the censuses of Iceland in 1703 and of Norway in 1801 do not provide such information, we have assumed that these regions were predominantly rural. However, the Norwegian city region of Christiania was treated as urban.

6 Historical longitudinal databases of household structures based on population registers from various parts of Europe were not included because converting them into a cross-sectional matrix of the NAPP/Mosaic data structure would require generalised solutions that are currently hard or impossible to obtain. Given that many of these sources are highly localised (e.g. Matthijs and Moreels 2010), they also have little added value for the geographic extension of our analysis.

7 The median proportion of the aged in our dataset is 5.33%, but the proportions range from less than 1% to almost 11%.

8 As this measure includes both elderly people who were living in one-person households and those who were sharing domestic space with other people in the absence of any consanguineal or affinal ties (i.e. living as unrelated lodgers or inmates), it combines primary and secondary unrelated individuals (Kobrin 1976; Ruggles 1988; Wall 1984). While this measure is based on a less conservative definition of “living alone” than the one that is commonly used in contemporary demography (e.g. Tomassini et al. 2004), it better accounts for historical realities. For the elderly in these societies, being a secondary unrelated individual, like living alone, was usually associated with comparable socio-economic deprivation and life course insecurity (Wall 1984; Szołtysek 2015, ch. 10.9; Modell and Hareven 1973).

9 The Moran’s *I* values were obtained by the Monte Carlo simulation procedure implemented in the R library *spdep* (Bivand and Piras 2015).

10 The *p* values for the LISA statistics were obtained through the analytical approach. Like the permutation approach, this procedure is not free from the multiple comparisons bias or from potential correlation between locations with common neighbours. Thus, we used the *p* values adjustments provided within the *spdep* package (Bivand and Piras 2015). For the sensitivity analysis, two alternative methods were used: the Bonferroni correction and the false discovery rate (Castro and Singer 2006). The two methods returned identical outcomes.

11 For technical details on the computation of geocovariates, see Electronic Supplementary Material 2.

12 We used this measure as a convenient shorthand for two separate, but also crude measures of fertility and mortality (child/woman ratios and per cent elderly) used in earlier literature (Ruggles 2009, 2010; Gruber and Szołtysek 2012).

13 For territories not covered by the Princeton data (such as Turkey or Albania) we used indicators derived from more recent literature (e.g. Falkingham and Gjonça 2001).

14 Our measure of age-heaping is the Total Modified Whipple’s Index (Spoorenberg 2007), which takes account of the preference for and the avoidance of all 10 digits across the 23–62 age interval against the assumed linearity and rectangularity over a 5-year age range.

15 The index combines the proportion of boys among the last children (aged 10–14 and living in households headed by their parents) and the surplus of boys over 100 girls in the 0–4 age group. In our dataset, the index takes values from zero to 14, where zero indicates no preference for sons in the households and in the whole population, and 14 indicates a very strong preference for sons. Given the highly skewed distribution of the index across our data (185 of populations have values equalling zero), the continuous index was transformed for the regression analysis into a discrete variable with three levels: lack of son preference, son preference index between one and three, and son preference equalling four or higher. For further details, see Gruber and Szołtysek (2016, 142–143).

16 More than 83% of the locations contribute to the positive spatial autocorrelation, with 33% of the locations lying in the *high–high* quadrant of the so-called Moran scatterplot (Anselin 1995; available upon request), which includes regions with a high value of the variable surrounded by regions with high values, and the remaining 50% of the locations lying in the *low–low* quadrant (low value regions surrounded by other regions with low values). The *low–high* and the *high–low* quadrants comprise 7.94% and 8.30% of the observations, respectively.

17 Out of the six populations in our data in which 62% or more of the elderly were living in stem-family-like constellations, three are early populations in Germany, and the other three are late nineteenth-century populations in central Ukraine.

18 Only two out of the 86 regions from the 1881 Great Britain census have less than 10% of the LMD variable, but none have the null value. Sixteen non-British regions in the dataset displayed values of this variable that were lower than the lowest English score. Seven of these regions were in Sweden, and their populations were surveyed in nearly the same year.

19 Of the regions observed, 15.88% are in the *high–high* quadrant, almost 78% are in the *low–low* quadrant, 3.61% are in the *low–high* quadrant, and 2.53% are in the *high–low* quadrant.

20 Of the data points, 45.13% are in the *high–high* quadrant, 35.38% are in the *low–low* quadrant, 10.83% are in the *low–high* quadrant, and 8.66% are in the *high–low* quadrant.

21 The local Moran’s *I* is calculated in combination with a particular neighbourhood. Thus, according to the spatial weights matrix, the spatial range of the clusters should be seen in the broader context of the region, that is, including the neighbouring regions that are not highlighted (Anselin 2005).

22 Regression results are represented as standardised coefficients.

23 Although some of the variables used in the OLS models are correlated, in most cases the variance inflation factor (VIF) is below five and never exceeds eight. This suggests that there is no severe collinearity problem.
