# Supplementary material for: Making a Place for Space: A Demographic Spatial Perspective on Living Arrangements Among the Elderly in Historical Europe
Source: Eur J Popul. 2019 Mar 21;36(1):85–117. doi: 10.1007/s10680-019-09520-5 (PMC7018862; doi:10.1007/s10680-019-09520-5)
Supplement: Supplementary file 1 — Supplementary material 1 (PDF 668 kb) [file 10680_2019_9520_MOESM1_ESM.pdf]

Electronic Supplementary Material 1

Table 1A Mosaic and NAPP data used for analysis, with data references

Population	Region	Period	LMD	LLK	LWR	Population size
AL/Berat 1918	Balkans	after 1850	0.271	0.483	0.038	7424
AL/Durresi (city) 1918	Balkans	after 1850	0.431	0.314	0.051	4307
AL/Elbasani (city) 1918	Balkans	after 1850	0.372	0.261	0.082	10237
AL/Gora 1918	Balkans	after 1850	0.343	0.496	0.015	11298
AL/Kavaja (city) 1918	Balkans	after 1850	0.416	0.305	0.042	5522
AL/Kruja (city) 1918	Balkans	after 1850	0.375	0.335	0.081	3893
AL/Kruja 1918	Balkans	after 1850	0.335	0.549	0.017	4276
AL/Puka 1918	Balkans	after 1850	0.303	0.613	0.003	5008
AL/Shkodra (city) 1918	Balkans	after 1850	0.316	0.214	0.071	23590
AL/Shkodra 1918	Balkans	after 1850	0.311	0.555	0.046	12340
AL/Tirana (city) 1918	Balkans	after 1850	0.383	0.319	0.070	10416
AL/Tirana North 1918	Balkans	after 1850	0.270	0.548	0.047	14529
AL/Tirana South 1918	Balkans	after 1850	0.415	0.207	0.042	12206
AL/Zhuri 1918	Balkans	after 1850	0.290	0.547	0.024	15565
AT/Styria 1910	Habsburg	after 1850	0.221	0.020	0.267	6693
AT/Tyrol 1910	Habsburg	after 1850	0.179	0.070	0.150	6514
AT/Upper Austria 1910	Habsburg	after 1850	0.158	0.008	0.267	1675
AT/Waidhofen/Ybbs (city) 1910	Habsburg	after 1850	0.167	0.037	0.321	5154
BE/Western Flanders 1814	West	1800-1850	0.173	0.009	0.185	13666
BG/Čepelare (city) 1880-1947	Balkans	after 1850	0.574	0.049	0.090	1783
BG/Rhodope region 1877-1947	Balkans	after 1850	0.624	0.093	0.023	6590
CH/Zürich (city) 1870	West	after 1850	0.130	0.018	0.373	8152
CH/Zürich North rural 1671-1685	West	before 1800	0.288	0.006	0.074	6400
CH/Zürich South rural 1678-1762	West	before 1800	0.379	0.079	0.050	4588
DE/Arnsberg 1846	Germany	1800-1850	0.337	0.010	0.192	2111
DE/Braunschweig 1846	Germany	1800-1850	0.008	0.000	0.322	2014
DE/Danzig and Posen 1858	Germany	after 1850	0.183	0.024	0.256	3468
DE/Düsseldorf 1846	Germany	1800-1850	0.374	0.043	0.104	2185
DE/Höhscheid 1846	Germany	1800-1850	0.067	0.004	0.192	6306
DE/Koblenz 1846	Germany	1800-1850	0.215	0.011	0.118	2092
DE/Liegnitz 1846	Germany	1800-1850	0.288	0.014	0.253	2607
DE/Mecklenburg-Schwerin Northeast 1819	Germany	1800-1850	0.221	0.011	0.219	9602

DE/Mecklenburg-Schwerin Northeast 1867	Germany	after 1850	0.378	0.039	0.141	14107
DE/Mecklenburg-Schwerin Northwest 1819	Germany	1800-1850	0.181	0.011	0.213	2061
DE/Mecklenburg-Schwerin Northwest 1867	Germany	after 1850	0.358	0.005	0.221	3914
DE/Mecklenburg-Schwerin Southeast 1867	Germany	after 1850	0.467	0.046	0.171	3232
DE/Mecklenburg-Schwerin Southwest 1819	Germany	1800-1850	0.344	0.008	0.169	10209
DE/Mecklenburg-Schwerin Southwest 1867	Germany	after 1850	0.484	0.022	0.138	16025
DE/Meppen 1749	Germany	before 1800	0.625	0.019	0.056	12360
DE/Meppen and Cloppenburg 1700	Germany	before 1800	0.482	0.012	0.119	8607
DE/Merseburg 1846	Germany	1800-1850	0.086	0.000	0.276	1939
DE/Münster 1846	Germany	1800-1850	0.494	0.036	0.024	1278
DE/Rheine-Bevergern 1700	Germany	before 1800	0.733	0.034	0.043	3506
DE/Rheine-Bevergern 1749	Germany	before 1800	0.640	0.015	0.076	5558
DE/Rostock (city) 1900	Germany	after 1850	0.159	0.024	0.313	55705
DE/Sachsen-Coburg 1846	Germany	1800-1850	0.366	0.020	0.109	2096
DE/Sachsen-Gotha 1846	Germany	1800-1850	0.216	0.010	0.186	2284
DE/Sigmaringen 1861	Germany	after 1850	0.291	0.031	0.174	6541
DE/Stromberg 1749	Germany	before 1800	0.400	0.038	0.190	16251
DE/Trier 1846	Germany	1800-1850	0.143	0.000	0.310	1712
DE/urban Centre 1846	Germany	1800-1850	0.195	0.019	0.247	3051
DE/urban East 1846	Germany	1800-1850	0.117	0.010	0.243	2892
DE/urban West 1846	Germany	1800-1850	0.126	0.009	0.260	4193
DE/Vechta 1700	Germany	before 1800	0.446	0.012	0.080	10897
Denmark/Aalborg 1787	Scandinavia	before 1800	0.224	0.029	0.183	42674
Denmark/Aarhus 1787	Scandinavia	before 1800	0.294	0.034	0.232	23703
Denmark/Bornholm 1787	Scandinavia	before 1800	0.266	0.024	0.150	17674
Denmark/Frederiksborg 1787	Scandinavia	before 1800	0.263	0.028	0.183	41842
Denmark/Hjørring 1787	Scandinavia	before 1800	0.230	0.021	0.171	40791
Denmark/Holbæk 1787	Scandinavia	before 1800	0.222	0.025	0.145	44388
Denmark/København 1787	Scandinavia	before 1800	0.162	0.021	0.349	111301
Denmark/Maribo 1787	Scandinavia	before 1800	0.224	0.025	0.206	47937
Denmark/Odense 1787	Scandinavia	before 1800	0.258	0.037	0.224	59432
Denmark/Præstø 1787	Scandinavia	before 1800	0.237	0.025	0.182	49364
Denmark/Randers 1787	Scandinavia	before 1800	0.270	0.030	0.220	45400
Denmark/Ribe 1787	Scandinavia	before 1800	0.383	0.031	0.162	31248

Denmark/Ringkøbing 1787	Scandinavia	before 1800	0.288	0.027	0.168	34964
Denmark/Roskilde 1787	Scandinavia	before 1800	0.254	0.031	0.152	21195
Denmark/Skanderborg 1787	Scandinavia	before 1800	0.218	0.021	0.211	25094
Denmark/Sorø 1787	Scandinavia	before 1800	0.187	0.017	0.219	39403
Denmark/Svendborg 1787	Scandinavia	before 1800	0.238	0.028	0.196	51049
Denmark/Thisted 1787	Scandinavia	before 1800	0.274	0.024	0.148	27438
Denmark/Tønder 1787	Scandinavia	before 1800	0.365	0.054	0.166	8887
Denmark/Vejle 1787	Scandinavia	before 1800	0.309	0.036	0.178	35611
Denmark/Viborg 1787	Scandinavia	before 1800	0.260	0.036	0.168	39228
England/Bedfordshire 1881	Great Britain	after 1850	0.137	0.060	0.221	15167
England/Berkshire 1881	Great Britain	after 1850	0.136	0.033	0.218	24837
England/Buckinghamshire 1881	Great Britain	after 1850	0.144	0.028	0.260	15686
England/Cambridgeshire 1881	Great Britain	after 1850	0.123	0.038	0.237	18797
England/Cheshire 1881	Great Britain	after 1850	0.204	0.056	0.236	62469
England/Cornwall 1881	Great Britain	after 1850	0.177	0.034	0.257	32580
England/Cumberland 1881	Great Britain	after 1850	0.164	0.038	0.210	24890
England/Derbyshire 1881	Great Britain	after 1850	0.205	0.028	0.215	38473
England/Devonshire 1881	Great Britain	after 1850	0.157	0.037	0.282	59927
England/Dorset 1881	Great Britain	after 1850	0.129	0.034	0.255	20061
England/Durham 1881	Great Britain	after 1850	0.227	0.038	0.214	86722
England/Essex 1881	Great Britain	after 1850	0.138	0.036	0.273	54628
England/Gloucestershire 1881	Great Britain	after 1850	0.131	0.045	0.302	51885
England/Hampshire 1881	Great Britain	after 1850	0.162	0.035	0.271	56385
England/Herefordshire 1881	Great Britain	after 1850	0.125	0.030	0.311	11762
England/Hertfordshire 1881	Great Britain	after 1850	0.178	0.025	0.221	19927
England/Huntingdonshire 1881	Great Britain	after 1850	0.083	0.029	0.367	5351
England/Kent 1881	Great	after 1850	0.147	0.049	0.276	97852

	Britain					
England/Lancashire 1881	Great Britain	after 1850	0.248	0.053	0.224	345052
England/Leicestershire 1881	Great Britain	after 1850	0.162	0.043	0.238	32554
England/Lincolnshire 1881	Great Britain	after 1850	0.133	0.036	0.241	45612
England/Middlesex 1881	Great Britain	after 1850	0.145	0.043	0.371	290788
England/Norfolk 1881	Great Britain	after 1850	0.125	0.034	0.296	44172
England/Northamptonshire 1881	Great Britain	after 1850	0.141	0.022	0.257	27371
England/Northumberland 1881	Great Britain	after 1850	0.184	0.057	0.179	43428
England/Nottinghamshire 1881	Great Britain	after 1850	0.202	0.039	0.224	43689
England/Oxford 1881	Great Britain	after 1850	0.115	0.046	0.220	18088
England/Shropshire 1881	Great Britain	after 1850	0.143	0.041	0.228	26324
England/Somerset 1881	Great Britain	after 1850	0.143	0.037	0.261	48327
England/Staffordshire 1881	Great Britain	after 1850	0.211	0.040	0.266	100644
England/Suffolk 1881	Great Britain	after 1850	0.134	0.037	0.246	34818
England/Surrey 1881	Great Britain	after 1850	0.149	0.041	0.402	146492
England/Sussex 1881	Great Britain	after 1850	0.135	0.039	0.327	49328
England/Warwick 1881	Great Britain	after 1850	0.182	0.037	0.274	73402
England/Westmorland 1881	Great Britain	after 1850	0.101	0.023	0.265	6392
England/Wiltshire 1881	Great Britain	after 1850	0.137	0.022	0.277	24680
England/Worcestershire 1881	Great Britain	after 1850	0.160	0.044	0.248	37557
England/Yorkshire 1881	Great Britain	after 1850	0.197	0.044	0.218	288108
ES/Barcelona province urban 1889	West	after 1850	0.471	0.047	0.075	7001
ES/Catalonia rural 1880-1890	West	after 1850	0.464	0.022	0.148	7115
ES/other Catalonia urban 1889	West	after 1850	0.370	0.027	0.139	9881
F/Northeast 1846	West	1800-1850	0.172	0.015	0.203	4444

F/Northwest 1846	West	1800-1850	0.249	0.050	0.223	5914
F/South 1846	West	1800-1850	0.413	0.026	0.162	6609
F/Southwest 1831-1901	West	after 1850	0.460	0.031	0.181	5109
F/St. Emilion (city) 1846/1856	West	after 1850	0.447	0.016	0.188	5669
H/Great Plain 1869	Habsburg	after 1850	0.186	0.059	0.192	3781
H/North-East 1869	Habsburg	after 1850	0.342	0.048	0.312	2072
H/Northern Transdanubia 1869	Habsburg	after 1850	0.422	0.092	0.146	4067
H/Southern Transdanubia 1869	Habsburg	after 1850	0.295	0.157	0.225	3804
HR/Dubrovnik area 1674	Habsburg	before 1800	0.587	0.129	0.026	1880
Iceland 1703	Scandinavia	before 1800	0.151	0.025	0.487	51003
Islands in the British Seas/Guernsey and Alderney 1881	Great Britain	after 1850	0.150	0.042	0.269	33693
Islands in the British Seas/Isle of Man 1881	Great Britain	after 1850	0.177	0.046	0.220	53466
Islands in the British Seas/Jersey 1881	Great Britain	after 1850	0.188	0.061	0.254	52455
LT/Kovno 1847	East	1800-1850	0.459	0.135	0.041	9954
LT/Vilna 1847	East	1800-1850	0.544	0.219	0.030	9963
LV/Courland Goldingen+Pilten 1797	East	before 1800	0.326	0.088	0.230	13377
LV/Courland Mitau 1797	East	before 1800	0.231	0.161	0.175	10490
LV/Courland Selburg 1797	East	before 1800	0.380	0.143	0.226	7200
LV/Courland Tuckum 1797	East	before 1800	0.156	0.033	0.301	4740
NL/Eindhoven and Helmond (city) 1811	West	1800-1850	0.153	0.022	0.185	4774
NL/Goes (city) 1810	West	1800-1850	0.038	0.000	0.435	4350
NL/North Brabant 1810	West	1800-1850	0.169	0.083	0.169	6488
NL/Tilburg (city) 1811	West	1800-1850	0.061	0.029	0.266	9679
NL/Zeeland 1811	West	1800-1850	0.065	0.000	0.323	14746
Norway/Aggershuus 1801	Scandinavia	1800-1850	0.291	0.040	0.258	56761
Norway/Bratsberg 1801	Scandinavia	1800-1850	0.274	0.029	0.196	47503
Norway/Buskerud 1801	Scandinavia	1800-1850	0.262	0.055	0.235	58184
Norway/Christiania 1801	Scandinavia	1800-1850	0.118	0.025	0.485	9211
Norway/Christians 1801	Scandinavia	1800-1850	0.348	0.035	0.250	71555
Norway/Finmarken 1801	Scandinavia	1800-1850	0.257	0.033	0.156	26925
Norway/Hedemarken 1801	Scandinavia	1800-1850	0.371	0.045	0.224	61025
Norway/Jarlsberg O 1801	Scandinavia	1800-1850	0.266	0.025	0.243	39837
Norway/Lister Og M 1801	Scandinavia	1800-1850	0.331	0.042	0.171	39570
Norway/Nedenæs 1801	Scandinavia	1800-1850	0.386	0.031	0.157	30997
Norway/Nordland 1801	Scandinavia	1800-1850	0.273	0.015	0.194	51455
Norway/Nordre Berg 1801	Scandinavia	1800-1850	0.455	0.043	0.168	52601

Norway/Nordre Tron 1801	Scandinavia	1800-1850	0.250	0.022	0.200	43443
Norway/Romsdal 1801	Scandinavia	1800-1850	0.403	0.025	0.240	57710
Norway/Smaalehnene 1801	Scandinavia	1800-1850	0.229	0.039	0.259	50141
Norway/Søndre Berg 1801	Scandinavia	1800-1850	0.295	0.029	0.243	78768
Norway/Søndre Tron 1801	Scandinavia	1800-1850	0.325	0.024	0.193	51272
Norway/Stavanger 1801	Scandinavia	1800-1850	0.334	0.033	0.206	42268
Norway/Trondheim 1801	Scandinavia	1800-1850	0.100	0.003	0.474	8847
PL/Central Belarus 1768-1804	East	before 1800	0.466	0.271	0.019	19176
PL/Chelmska Land 1791-1792	East	before 1800	0.547	0.168	0.080	25193
PL/Greater Poland 1666-1809	East	before 1800	0.349	0.047	0.198	5763
PL/Kujavia 1766-1792	East	before 1800	0.391	0.019	0.138	13320
PL/Lesser Poland 1789-1792	East	before 1800	0.393	0.005	0.194	14371
PL/Ostrzeszow County 1790-1791	East	before 1800	0.407	0.010	0.180	8358
PL/Podolia 1785-1819	East	before 1800	0.471	0.059	0.196	5526
PL/Polesia 1795	East	before 1800	0.399	0.492	0.010	25332
PL/Silesia 1747-1805	East	before 1800	0.508	0.010	0.144	12265
PL/Warmia 1695-1772	East	before 1800	0.056	0.000	0.157	2543
PL/Wielunskie County 1790-1792	East	before 1800	0.468	0.005	0.115	9945
PL/Zhytomyr County 1791	East	before 1800	0.416	0.375	0.040	14026
RO/Eastern Wallachia 1838	Balkans	1800-1850	0.190	0.011	0.032	5089
RO/Moldavia Catholics 1781-1787	Balkans	before 1800	0.324	0.000	0.216	1992
RO/Moldavia Catholics 1866-1879	Balkans	after 1850	0.217	0.011	0.054	3299
RO/Northern Wallachia 1838	Balkans	1800-1850	0.152	0.011	0.148	5806
RO/Partium 1869	Habsburg	after 1850	0.519	0.078	0.140	3471
RO/Southern Wallachia 1838	Balkans	1800-1850	0.144	0.004	0.107	5411
RO/Southwestern Wallachia 1838	Balkans	1800-1850	0.211	0.055	0.036	5240
RO/Transylvania 1869	Habsburg	after 1850	0.366	0.013	0.074	5801
RUS/Braclav Governorate 1795	East	before 1800	0.401	0.117	0.044	8050
RUS/Gagarin villages 1814	East	1800-1850	0.439	0.474	0.000	2955
RUS/Moscow area 1897	East	after 1850	0.519	0.314	0.074	11559
RUS/Ural Centre 1710	East	before 1800	0.450	0.386	0.005	4213
RUS/Ural East 1710	East	before 1800	0.397	0.357	0.032	2723
RUS/Ural iron plants 1710	East	before 1800	0.465	0.116	0.000	1471
RUS/Ural North 1710	East	before 1800	0.425	0.353	0.007	5253
RUS/Ural North Centre 1710	East	before 1800	0.418	0.423	0.000	3886
RUS/Ural South 1710	East	before 1800	0.450	0.305	0.020	4713
RUS/Ural Verkhoturye (city) 1710	East	before 1800	0.500	0.100	0.067	1911
RUS/Ural West 1710	East	before 1800	0.407	0.368	0.011	3566

Scotland/Aberdeenshire 1881	Great Britain	after 1850	0.124	0.027	0.254	268025
Scotland/Angus and Forfarshire 1881	Great Britain	after 1850	0.139	0.032	0.254	266383
Scotland/Argyll 1881	Great Britain	after 1850	0.141	0.050	0.204	78491
Scotland/Ayr 1881	Great Britain	after 1850	0.149	0.033	0.241	217561
Scotland/Banff 1881	Great Britain	after 1850	0.129	0.026	0.222	63003
Scotland/Berwickshire 1881	Great Britain	after 1850	0.135	0.041	0.213	35401
Scotland/Bute 1881	Great Britain	after 1850	0.120	0.066	0.239	17642
Scotland/Caithness 1881	Great Britain	after 1850	0.132	0.037	0.183	38834
Scotland/Clackmannanshire 1881	Great Britain	after 1850	0.131	0.041	0.235	25674
Scotland/Dumfriesshire 1881	Great Britain	after 1850	0.129	0.044	0.214	64268
Scotland/Dunbartonshire 1881	Great Britain	after 1850	0.164	0.044	0.254	75389
Scotland/Fife 1881	Great Britain	after 1850	0.118	0.031	0.246	171955
Scotland/Inverness-shire 1881	Great Britain	after 1850	0.146	0.046	0.189	89194
Scotland/Kincardinshire 1881	Great Britain	after 1850	0.110	0.029	0.249	34471
Scotland/Kinross-shire 1881	Great Britain	after 1850	0.093	0.019	0.268	7466
Scotland/Kirkudbrightshire 1881	Great Britain	after 1850	0.134	0.034	0.220	42153
Scotland/Lanarkshire 1881	Great Britain	after 1850	0.191	0.036	0.255	939368
Scotland/Mid Lothian and Edinburghshire 1881	Great Britain	after 1850	0.154	0.040	0.275	427945
Scotland/Morayshire and Elginshire 1881	Great Britain	after 1850	0.118	0.029	0.243	44020
Scotland/Nairnshire 1881	Great Britain	after 1850	0.114	0.040	0.235	9552
Scotland/Orkney 1881	Great Britain	after 1850	0.200	0.045	0.177	32043
Scotland/Peeblesshire 1881	Great Britain	after 1850	0.115	0.064	0.194	14265
Scotland/Perthshire 1881	Great Britain	after 1850	0.110	0.035	0.253	128350

Scotland/Renfrewshire 1881	Great Britain	after 1850	0.180	0.040	0.248	225516
Scotland/Ross and Cromarty 1881	Great Britain	after 1850	0.168	0.040	0.175	78745
Scotland/Roxburghshire 1881	Great Britain	after 1850	0.153	0.038	0.200	53450
Scotland/Selkirk 1881	Great Britain	after 1850	0.194	0.035	0.165	25552
Scotland/Shetland 1881	Great Britain	after 1850	0.276	0.055	0.150	29735
Scotland/Stirlingshire 1881	Great Britain	after 1850	0.145	0.036	0.254	112640
Scotland/Sutherland 1881	Great Britain	after 1850	0.161	0.053	0.138	23401
Scotland/West Lothian and Linlithgowshire 1881	Great Britain	after 1850	0.135	0.028	0.268	43619
Scotland/Wigtown 1881	Great Britain	after 1850	0.138	0.048	0.221	38611
SE/Jasenica 1863	Balkans	after 1850	0.537	0.293	0.049	7128
SE/Jasenica 1884	Balkans	after 1850	0.476	0.395	0.040	9434
SE/Kruševac (city) 1863	Balkans	after 1850	0.611	0.000	0.167	2618
SK/Central 1869	Habsburg	after 1850	0.568	0.151	0.157	1779
SK/East 1869	Habsburg	after 1850	0.393	0.109	0.147	3601
SK/West 1869	Habsburg	after 1850	0.457	0.036	0.119	3030
Sweden/Älvsborg 1880	Scandinavia	after 1850	0.122	0.002	0.333	285095
Sweden/Blekinge 1880	Scandinavia	after 1850	0.030	0.001	0.439	140435
Sweden/Gävleborg 1880	Scandinavia	after 1850	0.379	0.009	0.243	179944
Sweden/Göteborg och Bohus 1880	Scandinavia	after 1850	0.148	0.010	0.334	267618
Sweden/Gotland 1880	Scandinavia	after 1850	0.425	0.022	0.210	55649
Sweden/Halland 1880	Scandinavia	after 1850	0.178	0.005	0.320	146051
Sweden/Jämtland 1880	Scandinavia	after 1850	0.166	0.004	0.348	88665
Sweden/Jönköping 1880	Scandinavia	after 1850	0.029	0.000	0.408	188050
Sweden/Kalmar 1880	Scandinavia	after 1850	0.035	0.001	0.397	252302
Sweden/Kopparberg 1880	Scandinavia	after 1850	0.432	0.033	0.188	191943
Sweden/Kristianstad 1880	Scandinavia	after 1850	0.015	0.001	0.433	225267
Sweden/Kronoberg 1880	Scandinavia	after 1850	0.014	0.000	0.386	161842
Sweden/Malmöhus 1880	Scandinavia	after 1850	0.016	0.000	0.422	363161
Sweden/Norrbotten 1880	Scandinavia	after 1850	0.342	0.010	0.268	91037
Sweden/Örebro 1880	Scandinavia	after 1850	0.107	0.002	0.371	184802
Sweden/Östergötland 1880	Scandinavia	after 1850	0.039	0.001	0.451	278266
Sweden/Skaraborg 1880	Scandinavia	after 1850	0.096	0.002	0.312	257417

Sweden/Södermanland 1880	Scandinavia	after 1850	0.082	0.002	0.447	142565
Sweden/Stockholm 1880	Scandinavia	after 1850	0.076	0.004	0.556	317514
Sweden/Uppsala 1880	Scandinavia	after 1850	0.198	0.004	0.400	139216
Sweden/Värmland 1880	Scandinavia	after 1850	0.246	0.010	0.283	271884
Sweden/Västerbotten 1880	Scandinavia	after 1850	0.231	0.003	0.320	107110
Sweden/Västernorrland 1880	Scandinavia	after 1850	0.119	0.000	0.370	164839
Sweden/Västmanland 1880	Scandinavia	after 1850	0.215	0.005	0.362	124153
TR/Istanbul (city) 1885	Balkans	after 1850	0.243	0.075	0.308	3408
TR/Istanbul (city) 1907	Balkans	after 1850	0.326	0.039	0.193	4946
UKR/Berdychiv (city) 1897	East	after 1850	0.359	0.087	0.214	11814
UKR/Berdychiv region North rural 1897	East	after 1850	0.689	0.058	0.071	3646
UKR/Berdychiv region North urban 1897	East	after 1850	0.736	0.055	0.032	2173
UKR/Berdychiv region South rural 1897	East	after 1850	0.658	0.064	0.097	2267
UKR/Berdychiv region South urban 1897	East	after 1850	0.476	0.175	0.063	2982
UKR/Hetmanate North 1765	East	before 1800	0.406	0.481	0.038	4046
UKR/Hetmanate Southeast 1765	East	before 1800	0.381	0.438	0.029	5479
UKR/Hetmanate Southwest 1765	East	before 1800	0.471	0.235	0.059	4564
UKR/Hetmanate West 1765	East	before 1800	0.451	0.377	0.049	4302
Wales/Anglesey 1881	Great Britain	after 1850	0.139	0.035	0.224	35091
Wales/Brecknockshire 1881	Great Britain	after 1850	0.148	0.032	0.253	54121
Wales/Caernarvonshire 1881	Great Britain	after 1850	0.147	0.034	0.222	123731
Wales/Cardiganshire 1881	Great Britain	after 1850	0.142	0.029	0.212	95062
Wales/Carmarthenshire 1881	Great Britain	after 1850	0.154	0.024	0.230	111149
Wales/Denbighshire 1881	Great Britain	after 1850	0.152	0.039	0.258	112824
Wales/Flintshire 1881	Great Britain	after 1850	0.147	0.030	0.232	45409
Wales/Glamorganshire 1881	Great Britain	after 1850	0.208	0.040	0.241	515189
Wales/Merionethshire 1881	Great Britain	after 1850	0.153	0.033	0.232	68285
Wales/Monmouthshire 1881	Great Britain	after 1850	0.174	0.038	0.267	233719
Wales/Montgomeryshire 1881	Great Britain	after 1850	0.164	0.035	0.231	76292
Wales/Pembrokeshire 1881	Great	after 1850	0.157	0.036	0.234	83653

	Britain					
Wales/Radnorshire 1881	Great Britain	after 1850	0.127	0.038	0.254	18540

Data references

Mosaic data:

Karl Kaser, Siegfried Gruber, Gentiana Kera, Enriketa Pandelejmoni. *1918 Census of Albania, Version 0.1* [SPSS file]. Graz, 2011.

Laboratory of Historical Demography (MPIDR). *1869 Census of Hungary, Version 1.0* [Mosaic Historical Microdata File]. www.censusmosaic.org, 2014.

Laboratory of Historical Demography (MPIDR). *1910 Census of Austria, Version 1.0* [Mosaic Historical Microdata File]. www.censusmosaic.org, 2014.

Familiekunde Vlaanderen and Laboratory of Historical Demography (MPIDR). *1814 Census of Western Flanders, Version 1.0* [Mosaic Historical Microdata File]. www.censusmosaic.org, 2014.

Ulf Brunnbauer. *Household registers of Rhodope region, Version 1.0* [Mosaic Historical Microdata File]. www.censusmosaic.org, 2014.

Laboratory of Historical Demography (MPIDR). ***Status Animarum for Lisac and Pridvorje****, Version 1.0* [Mosaic Historical Microdata File]. www.censusmosaic.org, 2015.

Danish Data Archive. *1803 Census of Schleswig and Holstein, Version 1.1* [Mosaic Historical Microdata File]. www.censusmosaic.org, 2012.

Laboratory of Historical Demography (MPIDR). *1846 Census of France, Version 1.0* [Mosaic Historical Microdata File]. www.censusmosaic.org, 2014.

University of Bordeaux. *1831 Census of Sallespisse, Version 1.2* [Mosaic Historical Microdata File]. www.censusmosaic.org, 2013.

University of Bordeaux. *1836 Census of Boulazac, Version 1.1* [Mosaic Historical Microdata File]. www.censusmosaic.org, 2012.

University of Bordeaux. *1841 Census of St. Jean de Luz, Version 1.1* [Mosaic Historical Microdata File]. www.censusmosaic.org, 2012.

University of Bordeaux. *1841 Census of Targon, Version 1.1* [Mosaic Historical Microdata File]. www.censusmosaic.org, 2012.

University of Bordeaux. *1876 Census of Boulazac, Version 1.1* [Mosaic Historical Microdata File]. www.censusmosaic.org, 2012.

University of Bordeaux. *1901 Census of Sauternes, Version 1.1* [Mosaic Historical Microdata File]. www.censusmosaic.org, 2012.

University of Bordeaux. ***1846 Census of Saint-Émilion, Version 1.2*** [Mosaic Historical Microdata File]. www.censusmosaic.org, 2014.

University of Bordeaux. *1856 Census of Saint-Émilion, Version 1.2* [Mosaic Historical Microdata File]. www.censusmosaic.org, 2014.

Laboratory of Historical Demography (MPIDR). *1846 German Customs Union Census, Version 2.1* [Mosaic Historical Microdata File]. www.censusmosaic.org, 2014.

Laboratory of Historical Demography (MPIDR). *1846 Census of Höhscheid, Version 1.0* [Mosaic Historical Microdata File]. www.censusmosaic.org, 2014.

Laboratory of Historical Demography (MPIDR). *1858 German Customs Union Census, Version 1.0* [Mosaic Historical Microdata File]. www.censusmosaic.org, 2014.

Laboratory of Historical Demography (MPIDR). *1861 Census of Haigerloch, Version 1.0* [Mosaic Historical Microdata File]. www.censusmosaic.org, 2014.

State Main Archive Schwerin, Laboratory of Historical Demography (MPIDR), and Department of Multimedia and Data Processing, University of Rostock. *1819 Census of Mecklenburg-Schwerin, Version 1.0* [Mosaic Historical Microdata File]. www.censusmosaic.org, 2016.

State Main Archive Schwerin, Laboratory of Historical Demography (MPIDR), and Department of Multimedia and Data Processing, University of Rostock. ***1819 Census of Rostock, Version 1.0*** [Mosaic Historical Microdata File]. www.censusmosaic.org, 2015.

State Main Archive Schwerin, Laboratory of Historical Demography (MPIDR), and Department of Multimedia and Data Processing, University of Rostock. *1867 Census of Mecklenburg-Schwerin, Version 1.0* [Mosaic Historical Microdata File]. www.censusmosaic.org, 2016.

State Main Archive Schwerin, Laboratory of Historical Demography (MPIDR), and Department of Multimedia and Data Processing, University of Rostock. *1867 Census of Rostock, Version 1.0* [Mosaic Historical Microdata File]. www.censusmosaic.org, 2015.

State Main Archive Schwerin, Laboratory of Historical Demography (MPIDR), and Department of Multimedia and Data Processing, University of Rostock. *1900 Census of Rostock, Version 1.0* [Mosaic Historical Microdata File]. Rostock, Germany: www.censusmosaic.org, 2013.

Laboratory of Historical Demography (MPIDR). *1749 Status Animarum of Münster, Version 1.0* [Mosaic Historical Microdata File]. www.censusmosaic.org, 2014.

Laboratory of Historical Demography (MPIDR). *1690-1713 Status Animarum of Oldenburger Münsterland, Version 1.0* [Mosaic Historical Microdata File]. www.censusmosaic.org, 2014.

Laboratory of Historical Demography (MPIDR). ***1811 Census of Zeeland, Version 1.0*** [Mosaic Historical Microdata File]. www.censusmosaic.org, 2015.

Laboratory of Historical Demography (MPIDR). *1810 Census of North Brabant, Version 1.0* [Mosaic Historical Microdata File]. www.censusmosaic.org, 2015.

Mikołaj Szołtysek (2012) CEURFAMFORM database, Version 23 [SPSS file]. Rostock.

Laboratory of Historical Demography (MPIDR). *1781-1879 Status Animarum in Moldavia, Version 1.0* [Mosaic Historical Microdata File]. www.censusmosaic.org, 2015.

Laboratory of Historical Demography (MPIDR). *1838 Census of Wallachia, Version 1.0* [Mosaic Historical Microdata File]. www.censusmosaic.org, 2014.

Laboratory of Historical Demography (MPIDR). *1710 Russian enumeration of Ural region, Version 1.0* [Mosaic Historical Microdata File]. www.censusmosaic.org, 2017.

Laboratory of Historical Demography (MPIDR). *1765 Rumyantsev census of Hetmanate region, Version 1.0* [Mosaic Historical Microdata File]. www.censusmosaic.org, 2017.

Laboratory of Historical Demography (MPIDR). *1795 Braclav Region Revision Lists, Version 1.0* [Mosaic Historical Microdata File]. www.censusmosaic.org, 2014.

Laboratory of Historical Demography (MPIDR). *1797 Revision lists of Courland, Version 1.0* [Mosaic Historical Microdata File]. www.censusmosaic.org, 2017.

Laboratory of Historical Demography (MPIDR). *1814 Russian list of inhabitants, Version 1.0* [Mosaic Historical Microdata File]. www.censusmosaic.org, 2014.

Laboratory of Historical Demography (MPIDR). ***1847 Lithuanian Estate Household Listings, Version 1.0*** [Mosaic Historical Microdata File]. www.censusmosaic.org, 2015.

Laboratory of Historical Demography (MPIDR). *1897 Russian census, Moscow region, Version 1.0* [Mosaic Historical Microdata File]. www.censusmosaic.org,2014.

Tomasz Jankowski. *1897 Russian Census, Berdychiv region, Version 1.0* [Mosaic Historical Microdata File]. www.censusmosaic.org, 2017.

Joel M. Halpern and Siegfried Gruber. *1863 Census of Jasenički srez, Serbia, Version 1.1* [Mosaic Historical Microdata File]. www.censusmosaic.org, 2012.

Joel M. Halpern and Siegfried Gruber. *1884 Census of Jasenički srez, Serbia, Version 1.1* [Mosaic Historical Microdata File]. www.censusmosaic.org, 2012.

Laboratory of Historical Demography (MPIDR). ***1880-1890 Local Censuses in Catalonia, Version 1.0*** [Mosaic Historical Microdata File]. www.censusmosaic.org, 2015.

Ulrich Pfister. *1634-1764 Soul listings of canton Zürich, Version 1.0* [Mosaic Historical Microdata File]. www.censusmosaic.org, 2017.

Vienna Database on European Family History. *1671-1685 Church listings of canton Zürich, Version 1.0* [Mosaic Historical Microdata File]. www.censusmosaic.org, 2017.

Vienna Database on European Family History. *1870 Census of Zürich, Version 1.0* [Mosaic Historical Microdata File]. www.censusmosaic.org, 2017.

Alan Duben. *1885 Census of Istanbul, Version 1.0* [Mosaic Historical Microdata File]. www.censusmosaic.org, 2014.

Alan Duben. *1907 Census of Istanbul, Version 1.0* [Mosaic Historical Microdata File]. www.censusmosaic.org, 2014.

Cezary Kuko, Radoslaw Poniat. *Censuses of the Civil-Military Order Commissions 1791–1792* [Polish Urban Sample].

NAPP data:

Minnesota Population Center. *North Atlantic Population Project: Complete Count Microdata. Version 2.3* [Machine-readable database]. Minneapolis: Minnesota Population Center, 2016.

- England and Wales 1881: K. Schürer and M. Woollard, National Sample from the 1881 Census of Great Britain [computer file], Colchester, Essex: History Data Service, UK Data Archive [distributor], 2003
- Scotland 1881: K. Schürer and M. Woollard, National Sample from the 1881 Census of Great Britain [computer file], Colchester, Essex: History Data Service, UK Data Archive [distributor], 2003.
- Denmark 1787: Nanna Floor Clausen, Danish National Archives. 1787 Census of Denmark, Version 1.0
- Iceland 1703: Ólöf Garðarsdóttir (University of Iceland) and National Archives of Iceland (NAI). 1703 Census of Iceland, Version 1.0.
- Norway 1801: The Digital Archive (The National Archive), University of Bergen, and the Minnesota Population Center. Census of Norway 1801, Version 1.0. Bergen, Norway: University of Bergen, 2011.
- Sweden 1880: The Swedish National Archives, Umeå University, and the Minnesota Population Center. National Sample of the 1880 Census of Sweden, Version 1.0. Minneapolis: Minnesota Population Center [distributor], 2014.

Electronic Supplementary Material 2: Construction of GIS-based covariates

In deriving the population potential variable, we used global population count raster data obtained from the History Database of the Global Environment (HYDE), Version 3.2 (NEAA 2016). These data are available in 10-year intervals from 1700-2000. We chose the data for 1800. It is important to note that these data are estimates. We cut the file to ensure that we were only considering populations living in areas located between a longitude of 60° west and 60° east, and a latitude of 20° and 80° north. We then re-projected the raster data to a Lambert Azimuthal Equal Area projection. The population potential measure gives population situated nearby more weight than population further apart (see Stewart and Warntz 1958). Thus, locations surrounded by areas with high population numbers have a higher population potential compared to locations surrounded by sparsely populated areas. We produced this measure using the stewart-command in the R-library SpatialPosition with the following specifications: span=100, 000; b=2; typefct= exponential. As the location for which we performed the calculation, we used for the Mosaic dataset the coordinates for the 1,692 Mosaic locations from which we derived the data for our 126 Mosaic regions. We generated the population potential for each location, and obtained from the outcomes a population-weighted value for the 126 Mosaic regions. For the NAPP regional data we used as coordinate the location of the raster point within the NAPP region that had the highest population density in 1800, according to the HYDE database. This was motivated by the fact that the population potential measure is very sensitive to the population in the immediate surroundings. Thus, we decided not to take the geographical or population-weighted centroids of the NAPP regions, which might have been situated in sparsely populated subareas of these regions.

Identical procedure was used to create cropland variable. Since raster cropland data available in the History Database of the Global Environment (HYDE Version 3.2; NEAA 2016) have 10-year temporal resolution and it’s well established in historical literature that socio-economic changes connected to the industrial and agrarian revolution could lead to important shifts in the land use, we have decided to compute crop variable based on the HYDE data for the period nearest to census describing demographical structure of the each Mosaic and NAPP population.

The data on terrain ruggedness were obtained from the GTOPO30 elevation raster dataset, which is a global digital elevation model with a horizontal grid spacing of 30 arc seconds (USGS 2016). To derive the information on terrain ruggedness, we used the Terrain Ruggedness Index (TRI) (Wilson et al. 2007). We did so by applying the focal function in the

R-library raster (the TRI-formula is provided in the help function of ‘terrain’ in the raster library). For the Mosaic locations, we generated the information for our set of 1,692 locations by considering the raster data within a circle with a diameter of 7.5 km centred on the location coordinates. Based on these data, we derived the population-weighted values for our 126 Mosaic regions. For the NAPP regions we had to use another approach, as we did not have location information for all of the settlements within a NAPP region. Here we faced the challenge that especially NAPP regions in Scandinavia were characterised by vast subareas with low population density. In order to avoid that our regional TRI values are dominated by information for such sparsely populated areas, we decided to only consider those areas of a NAPP region that had a population density above five persons per km² in 1800 (determined through a mask operation using the HYDE 3.2 population density raster dataset).

References:

NEAA [Netherlands Environmental Assessment Agency] (2016): HYDE [History Database of the Global Environment], Version 3.2. (beta). Bilthoven: NEAA. Retrieved September 11, 2016 from ftp://ftp.pbl.nl/hyde/hyde3.2/2016_beta_release/zip/ (files: 1800AD_pop.zip, 1800AD_lu.zip.

Stewart, J. Q., & Warntz, W. (1958). Macrogeography and social science. *Geographical Review, 48*, 167-184.

USGS [U.S. Geological Survey Center for Earth Resources Observation and Science] (2016): GTOPO30 Global 30 Arc-second Elevation. Sioux Falls SD: USGS. Retrieved August 31, 2016 from http://earthexplorer.usgs.gov/ (files: gt30e020n40, gt30e020n90, gt30w020n40, gt30w020n90, gt30w060n90).

Wilson, M.F.J., O'Connell, B., Brown, C., Guinan, J.C., & Grehan, A.J. (2007). Multiscale terrain analysis of multibeam bathymetry data for habitat mapping on the continental slope. *Marine Geodesy*, 30, 3-35.
